# Electroacupuncture Alleviates Cerebral Ischemia/Reperfusion Injury in Rats by Histone H4 Lysine 16 Acetylation-Mediated Autophagy

**DOI:** 10.3389/fpsyt.2020.576539

**Published:** 2020-12-18

**Authors:** Shu-Ying Xu, He-Qun Lv, Wen-Qian Li, Hao Hong, Yong-Jun Peng, Bing-Mei Zhu

**Affiliations:** ^1^Department of Acupuncture and Rehabilitation, Affiliated Hospital of Nanjing University of Chinese Medicine, Nanjing, China; ^2^Department of Acupuncture and Encephalopathy, Yancheng Hospital of Traditional Chinese Medicine, Yancheng, China; ^3^Regenerative Medicine Research Center, West China Hospital, Sichuan University, Chengdu, China

**Keywords:** HMOF, SIRT 1, autophagy, electroacupuncture, H4K16Ac

## Abstract

**Background:** Electroacupuncture (EA) treatment in ischemic stroke has been highlighted recently; however, the specific mechanism is still elusive. Autophagy is considered a new target for cerebral ischemia/reperfusion (I/R), but whether it plays a role of protecting or causing rapid cell apoptosis remains unclear. Studies have reported that the reduction in lysine 16 of histone H4 acetylation coheres with autophagy induction. The primary purpose of the study was to explore whether EA could alleviate I/R via autophagy-mediated histone H4 lysine 16 acetylation in the middle cerebral artery occlusion (MCAO) rat model.

**Methods:** One hundred and twenty male Sprague-Dawley rats were divided into five groups: control group, MCAO group, MCAO+EA group, MCAO+EA+hMOF siRNA group, and MCAO+EA+Sirt1 inhibitor group. EA was applied to “Baihui” (Du20) and “Renzhong” (Du26) at 5 min after modeling and 16 h after the first EA intervention. The structure and molecular markers of the rat brain were evaluated.

**Results:** EA significantly alleviated I/R injury by upregulating the expressions of Sirt1, Beclin1, and LC3-II and downregulating the expressions of hMOF and H4K16ac. In contrast, the Sirt1 inhibitor lowered the increase in Sirt1, Beclin1, and LC3-II and enhanced the level of hMOF and H4K16ac expressions associated with EA treatment. Besides, ChIP assay revealed that the binding of H4K16ac in the Beclin1 promoter region of the autophagy target gene was significantly raised in the MCAO+EA group and MCAO+EA+hMOF siRNA group.

**Conclusions:** EA treatment inhibited the H4K16ac process, facilitated autophagy, and alleviated I/R injury. These findings suggested that regulating histone H4 lysine 16 acetylation-mediated autophagy may be a key mechanism of EA at Du20 and Du26 to treat I/R.

## Introduction

Stroke is one of the leading causes of death and disability worldwide, leading to a heavy financial burden and mental stress. WHO data displayed that the stroke burden may rise from about 38 million disability-adjusted life years (DALYs) in 1990 to 61 million DALYs in 2020 globally (1). Ischemic stroke accounts for about 80–85% of all stroke cases that induce reperfusion and damage brain tissue (2, 3). Worse, it leads to physical defects and mental disorders. Meta-analysis shows that one-quarter of stroke survivors experienced poststroke anxiety and that one-third suffered from poststroke depression (4, 5). The neuropsychiatric sequelae of stroke may prevent the recovery process, affect quality of life, and cause caregiver's fatigue. Therefore, it is crippling. Unfortunately, the underlying mechanism of ischemic stroke is still unclear due to its complexity. Moreover, cerebral ischemia/reperfusion (CIR) injury exacerbates brain damage after ischemia. It refers to a pathological process in which neuronal apoptosis is further aggravated after blood perfusion is restored in a short period (6), including cell autophagy, inflammation, mitochondrial dysfunction, and oxidative stress (7, 8). However, few effective treatments could prevent CIR injury.

Acupuncture has a long history in ischemic stroke therapy, and “Baihui” (Du20) and “Renzhong” (Du26) are two classic acupoints for the treatment of the disease. Electroacupuncture (EA) is a new combination of acupuncture and modern electrotherapy. Previous studies have reported that EA is an apparent effective cure for cerebral ischemia tolerance (9, 10). However, the detailed mechanisms remain elusive. Some studies have manifested the affiliation between the effect of EA on cerebral ischemia and autophagy (11).

Active autophagy has been intensively observed in the ischemic penumbra (12), confirming that it has occurred in both cerebral ischemia physiological and pathological processes. Some studies have pointed out that autophagy plays a crucial role in providing additional energy and nutrients for ischemic cells (13, 14). Besides, researches have shown that autophagy induction of cerebral ischemia injury presents more functions on inhibiting neuronal damage and promoting neuronal survival than its blockade (15, 16). Several studies demonstrate that excessive autophagy could increase the risk of cell death (17, 18). Therefore, the role of autophagy in cerebral ischemia is not exact yet, and whether activation of autophagy will increase neuronal mortality or not remains controversial. By this dual mechanism of action, we believe autophagy has the potential to act on cerebral ischemia treatments. Here, we used autophagic markers LC3-II and Beclin1 to test the autophagic flux by western blot.

MOF is a member of the MYST family of histone acetyltransferases (HATs) (19), while hMOF is a 61-ortholog Drosophila MOF (20). It has been demonstrated that hMOF competes in DNA damage repair, cell cycle, and cell differentiation (21). Sirt1 is a member of the histone deacetylase (HDAC) protein family sirtuin and has been shown to regulate the multiple cellular stress responses (22). Histone acetylation is regulated by HATs and HDACs. It is one of the essential posttranslational modifications. Recent research has found that acetyltransferase (HATs) hMOF and histone deacetylase (HDACs) SIRT1 is a molecular histone switch on H4K16 acetylation, whose balancing effects regulate autophagy (23).

In summary, a correlation among EA, H4K16ac, and autophagy was found, while the mechanism is still unclear. Our purpose is to explore whether the effect of EA on autophagy is mediated by H4K16ac and to investigate its potential neuroprotective agent.

## Methods

### Animal Preparation

Adult male Sprague-Dawley (SD) rats (6–8 weeks old, 260 ± 10 g) were provided by the Nanjing Traditional Chinese Medicine University experimental animal center. One hundred and twenty rats were randomly divided into the following five groups: control group, middle cerebral artery occlusion (MCAO) group, MCAO+EA group, MCAO+EA+hMOF siRNA group, and MCAO+EA+Sirt1 inhibitor group (*n* = 24 for each group). All the rats were placed in an environment with a temperature of 24 ± 1°C, humidity of 50%, a cycle of light for 12 h/darkness for 12 h.

The study protocol was approved by the Animal Care and Use Committee of the First Affiliated Hospital of the Medical College at Nanjing Traditional Chinese Medicine University and strictly followed the guidelines of the Guide for the Care and Use of Laboratory Rats.

### MCAO Model

According to the Longa thread embolization method, the right middle cerebral artery (MCA) was blocked in rats to establish a modified acute focal CIR model (MCAO) (24). SD rats were anesthetized with 10% chloral hydrate (0.36 ml/100 g) intraperitoneal injection, and a laser Doppler flowmeter probe was inserted into the rat skull to the surface of the rat cortex to monitor cerebral blood flow. In the rat's supine position, a midline neck incision was made to separate the extracranial branch of the internal carotid artery of the rat, the pterygopalatine artery, and it was closed with a micro-arterial clip. Then the common carotid artery and internal carotid artery were clamped with a micro-arterial clip, the external carotid artery was cut with microsurgical scissors, and a 4/0 nylon wire with a diameter of 0.18 mm was inserted into the internal carotid artery from the external carotid artery incision and sent into the skull (about 18–20 mm); there is a clear sense of resistance at this time, and the cerebral blood flow monitored by the laser Doppler cerebral blood flow instrument suddenly drops to <15% before the modeling, which was successful. After 1 h, the nylon thread was retracted to the external carotid artery, which was the beginning of reperfusion.

### EA Stimulation

EA intervention was operated at 5 min and 16 h after the model was successfully established. Two acupuncture needles (0.25-mm outer diameter) were inserted into the Du20 acupoints (in the middle of the parietal bone) and Du26 (1 mm below the nasal tip, in the middle of the nasolabial fold) subcutaneously, which were indwelled for 30 min. The G6805-II EA apparatus (Shanghai Medical Electronic Machine Co., Ltd., Shanghai, China) was connected above the two needles after 5 min and 16 h of ischemia, with sparse waves at 3.85 Hz for 1.28 s and dense waves at 6.25 Hz for 2.08 s. The strength was 0.8–1.0 mA for 30 min.

### Lateral Ventricular Administration

After two EA treatments, the heads of the MCAO+EA+hMOF siRNA group and MCAO+EA+Sirt1 inhibitor group were fixed to the stereotype of the brain; the anterior fontanelle was marked. The puncture point was 1 mm to the right of the sagittal suture and 1.5 mm behind the coronal suture, and the depth of the injection was 1.5 mm. At high speed, the skull was drilled directly above the right ventricular injection site of the hMOF siRNA group and the Sirt1 inhibitor group, the microsyringe was inserted into the skull, and the configured hMOF siRNA and Entranster™-*in vivo* transfection reagent and the Sirt1 inhibitor nicotinamide were slowly injected with the configured hMOF siRNA. The injection rate was 0.5 L/min, and the injection volume was 20 L/n. The injection was completed, bone paraffin was applied, and the skin was sutured to complete the brain localization injection.

### Evaluation of the Neurobehavioral Deficit Score

Neurological deficits were scored in a single-blind design at 24 h following reperfusion. There were eight grades (0–7 scores). Scoring criteria were as follows: a score of 0, no asymmetrical activities; a score of 1, left forepaw cannot completely extend when lifting the tail; a score of 2, left forepaw disability; a score of 3, left forepaw tightly closed to the chest wall; a score of 4, turning left when free-running; a score of 5, left forepaw makes an act of pushing back; a score of 6, the rotation was surrounding the original point; and a score of 7, the left limb cannot support the body (25).

### Calculate Cerebral Infarction Volume

Twenty-four hours after reperfusion, the rat heads were quickly cut off, and the material was taken. After freezing, the brain was divided into 2-mm-thick brain slices and immersed in a 37/2% TT solution (TTC, Xinong Inc., Beijing, China) and incubated for 15 min. After TTC staining is completed, it was fixed, stored, photographed, and scanned, and the Ulead Photo Express 2.0 image analysis software was used to outline the cerebral infarction. Ischemic penumbra is measured with the medical injury measurement software, and the entire left hemisphere in each layer of brain slices is measured separately. Multiplying the area by 2 will get us the total tissue area; to get the infarct area, we multiply the total area of the left hemisphere by 2 and subtract the total area of the normal tissue; by multiplying infarct area with the thickness of the brain slice of 2 mm, we calculate the volume of each cerebral infarct tissue; and then by dividing the total volume of the left hemisphere by 2, we obtain the percentage of infarct volume.

### Hematoxylin–Eosin Staining

Brain tissues were cleaned and fixed with PBS to prepare paraffin sections, which were dehydrated with conventional ethanol and xylene. The tissues were soaked in paraffin and embedded, sliced, and baked. The paraffin sections were dewaxed and rehydrated, stained with hematoxylin for 10 min, and washed with water. Eosin staining was re-dyed for 3 min, and the slices were sealed with neutral resin and observed under a microscope.

### Western Blot

RIPA lysate was used to lyse the tissue and extract the supernatant by centrifugation; the protein concentration was determined by the BCA method; the same amount of protein sample (10 μl per well) was loaded, and the wavelength of the microplate reader was set at 562 nm for measurement. After electrophoresis, the target protein was transferred to PVDF membranes washed with TBST; and 5% skim milk was used to block at room temperature for 1 h. The primary antibody (Beclin1) was diluted 1:1,000, incubated for 1.5 h, and washed with TBST three times for 5 min each, and the secondary antibody was diluted 1:1,000, incubated at room temperature for 1.5 h, and washed with TBST four times for 5 min each. The ECL exposure solution was mixed according to a 1:1 (solution A:B) ratio, evenly covered the whole film, was reacted for 2 min, and was put into the exposure meter for exposure detection, and GAPDH was used as the internal reference for grayscale value comparison.

### Quantitative Real-Time Polymerase Chain Reaction (qRT-PCR)

Fresh brain tissues were taken, RNA was extracted according to the instructions of the RNA extraction kit, reverse transcription to cDNA was carried out according to the instructions of the reverse transcription kit, and qPCR was performed using cDNA as the template. The reaction system is as follows: 2 × SYBR Mix 5 μl, 0.5 μl of each primer, 10 × cDNA 1 μl, and ddH2O 10 μl. The reaction parameters were set as follows: 50°C 2 min, 95°C 2 min, 95°C 15 s, and 60°C 1 min, for 40 cycles. The primer sequences are shown below. With GAPDH as the internal reference gene, the relative expression of Beclin1 mRNA was calculated according to the 2–ΔΔCt method. The primer sequences were as follows: hMOF, 5′-GAGCATGAGGCGATCACCA-3′ and 5′-CCCATAGTCCTCCGGGAAAG-3′; Sirt1, 5′-TGTCATAGGTTAGGTGGCGAGT-3′ and 5′-AGGTGTTGGTGGCAACTCTGAT-3′; Beclin1, 5′-CGAGTGTCAGAACTACAAACGCT-3′ and 5′-CTCCTCCTCCAAGGCCAACT-3′; and Rat-GAPDH, 5′-GGCAAGTTCAACGGCACAG-3′ and 5′-CGCCAGTAGACTCCACGACAT-3′.

### ChIP-qPCR

Using 100–150 mg of rat brain tissue, follow the steps of the ChIP kit instructions. Briefly, first, use formaldehyde to perform DNA-protein cross-linking experiments; then perform nuclear preparation and chromatin fragmentation to obtain cross-linking chromatin preparation; the chromatin immunoprecipitation method was used; the chromatin is eluted from the antibody/Protein G Magnetic Beads and de-crosslinked. Finally, the spin column is purified, and DNA bound to the antibody of interest is obtained. Quantify DNA by quantitative fluorescence PCR. The primer for the promoter region of Beclin1 gene is shown as follows: forward 5′-GGCGATGGGAACTCTGGA-3′ and reverse 5′-CCCCGACGCTCTTCACCT-3′; forward 5′-CGTCAAGGCGTCACTTCTGG-3′ and reverse 5′-ACCTCCAGAGTTCCCATCGC-3′; and forward 5′-CGGGCGATGGGAACTCTG-3′ and reverse 5′-CCCGACGCTCTTCACCTC-3′. Each value was normalized to the percentage of input DNA by using IP/INPUT = 2CtInput DNA – Ct IP DN (26).

### Statistical Analysis

SPSS 22.0 was used for statistical analysis, data were expressed by *x* ±*s, t*-test was used, comparison between multiple groups was done by one-way ANOVA, and *P* < 0.05 indicated a significant difference.

## Results

### EA Improves Neurological Functions and Relieves Cerebral Infarction

**Figure 1:**
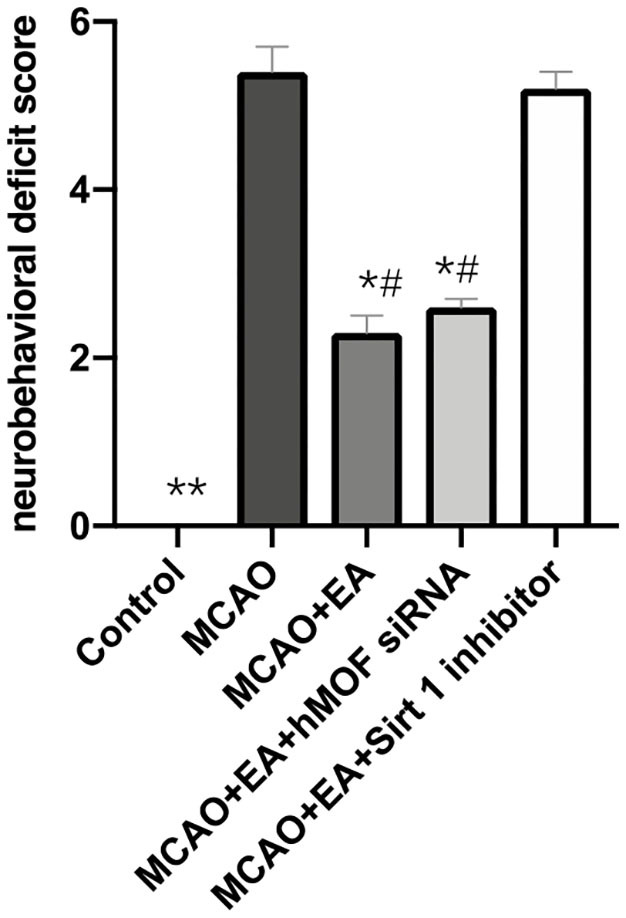
Electroacupuncture improves neurological scores. Data are presented as means ± SEM; *n* = 24 rats per group, *P < 0.01, **P < 0.001, vs. the MCAO group; ^#^*P* < 0.01, vs. the MCAO+EA+Sirt1 inhibitor group.

**Figure 2:**
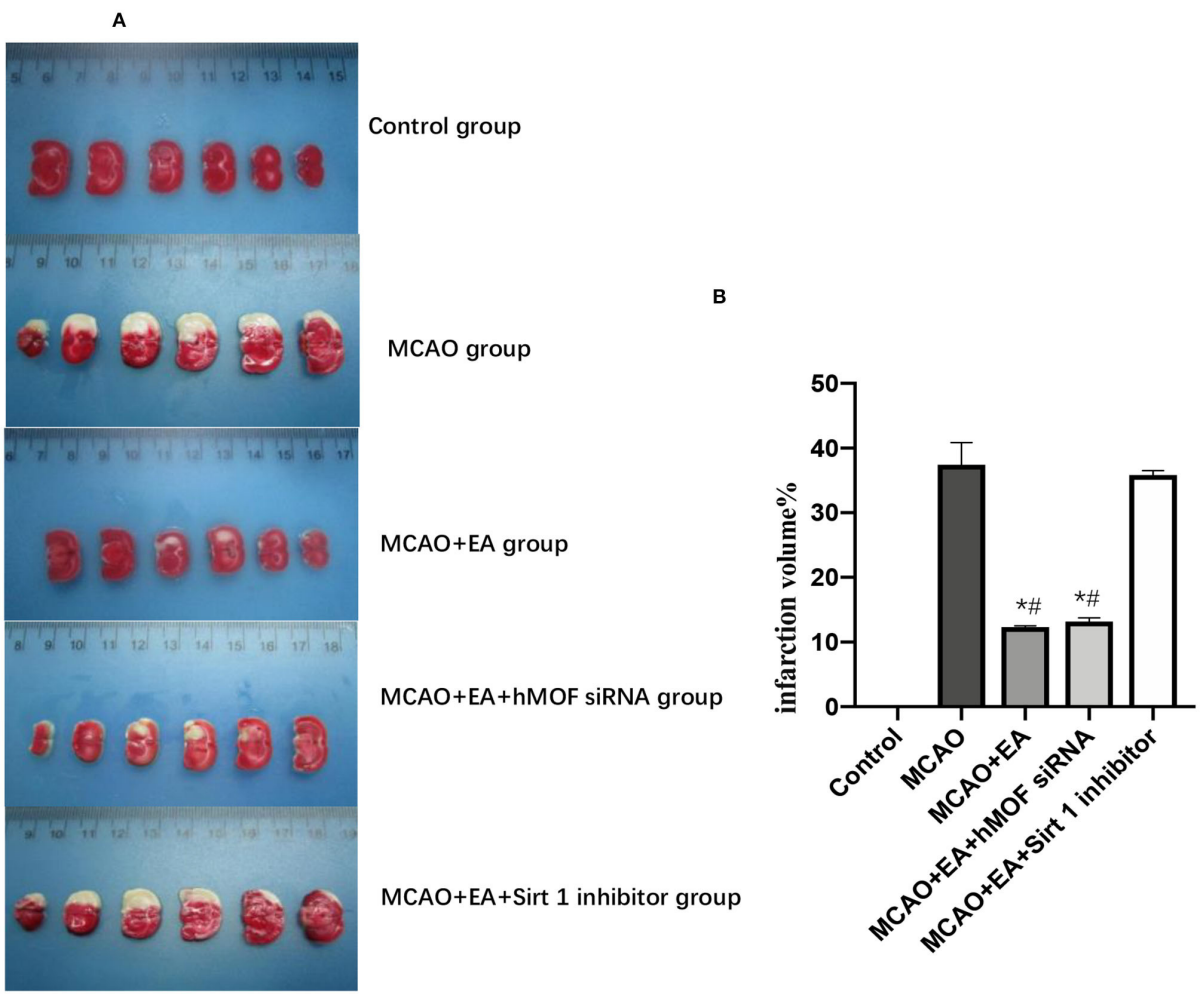
Image showing changes of infarct volume. **(A)** Typical photo micrographs of ischemic penumbra identified with TTC staining. **(B)** Calculation results of infarct volume; *n* = 6 per group. *P < 0.05, vs. the MCAO group; ^**#**^*P* < 0.05, vs. the MCAO+EA+Sirt1 inhibitor group.

As shown in Figure 1, the Longa test was used to evaluate the neurobehavioral function, and TTC staining was assessed to investigate cerebral infarction. Control group rats did not show any neurobehavioral impairment signs. In contrast, obvious white infarction areas were observed in the MCAO group (Figure 2A). As shown in Figure 1, the neurological scores of the MCAO+EA and MCAO+EA+hMOF siRNA groups were significantly decreased compared with those of the MCAO and MCAO+EA+Sirt1 inhibitor groups (*P* < 0.01). Simultaneously, when rats were treated with EA and hMOF siRNA, the infarct volume was obviously reduced compared to that in the MCAO group and MCAO+EA+Sirt1 inhibitor group (Figures 2A,B, *P* < 0.05). These results revealed that ischemia/reperfusion (I/R) and the Sirt1 inhibitor impaired neurologic function seriously, while the EA and hMOF inhibitor treatment alleviated it.

### EA Reduces the Pathological Damage to Right Striatum Brain Tissues in MCAO Rats

**Figure 3:**
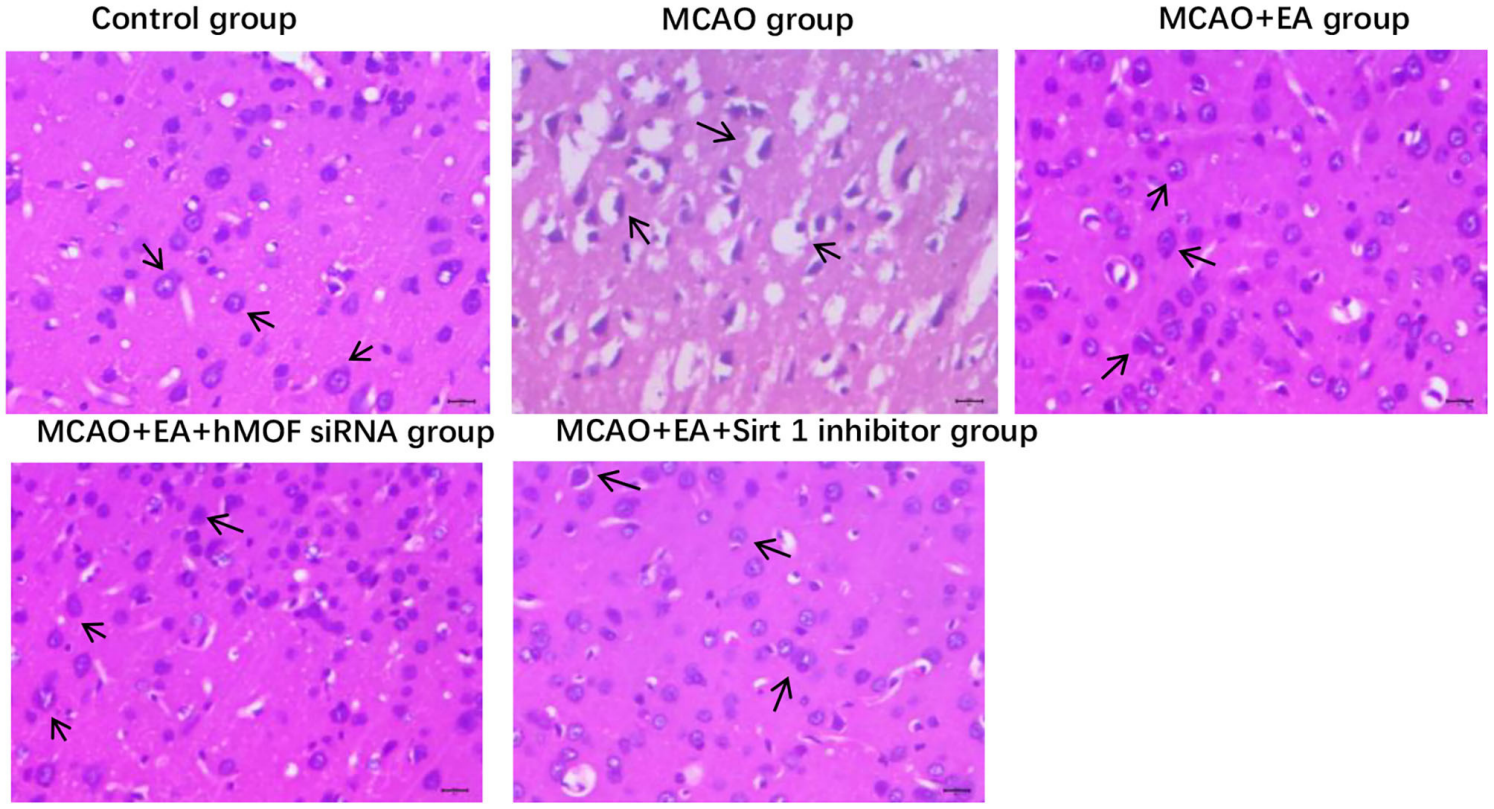
The histopathological findings in the right striatum brain tissue, ×400.

Hematoxylin–eosin staining showed significant differences in histopathological changes of parietal cortex neurons in each group (Figure 3). In the control group, the right striatum neurons have a neat, tidy structure, abundant cytoplasm, clear nuclei, and seamless interstitial edema. The MCAO+EA group and MCAO+EA+hMOF siRNA group displayed a few incomplete cell structures, resulting in shrinkage. Compared with the MCAO+EA group and MCAO+EA+hMOF siRNA group, the MCAO group and MCAO+EA+Sirt1 inhibitor group exhibited neuronal cell disorder, cell shrinkage, nuclear fragmentation, disappearance of the nuclear area, and large vacuoles.

### EA Alters hMOF, Sirt1, H4K16ac, LC3-II, and Beclin1 Expressions in the MCAO Rat Corpus Striatum

**Figure 4:**
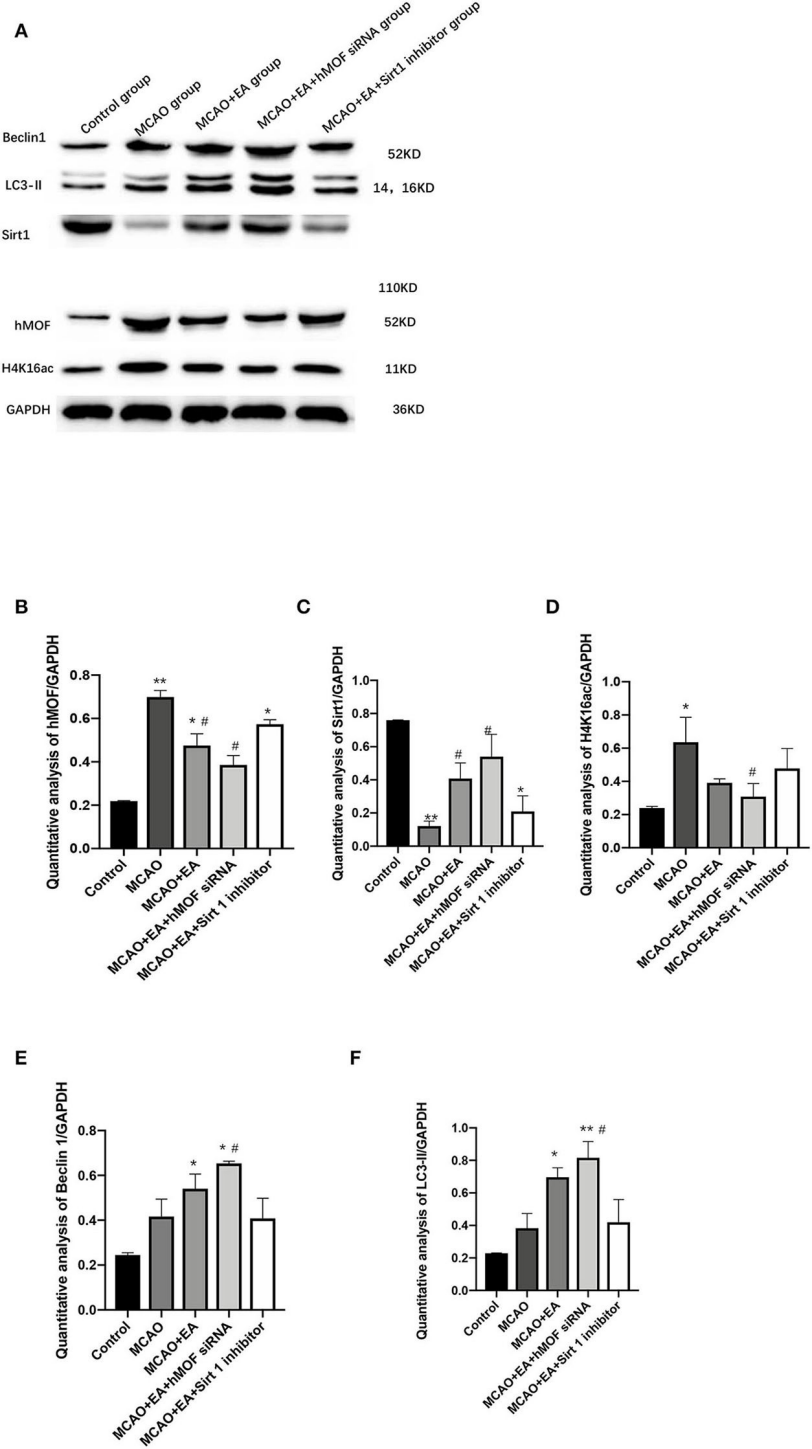
Electroacupuncture alters hMOF, Sirt1, H4K16ac, LC3-II, and Beclin1 expressions in the MCAO rat corpus striatum. **(A)** Western blot results of Sirt1, Beclin1, LC3-II, hMOF, H4K16ac, and GAPDH. **(B)** Quantification analysis of hMOF. Values are expressed as mean ± SEM (*n* = 6). *P < 0.05, **P < 0.01, vs. the control group; ^**#**^*P* < 0.05, vs. the MCAO group. **(C)** Quantification analysis of Sirt1. Values are expressed as mean ± SEM (*n* = 6). *P < 0.05, **P < 0.01, vs. the control group; ^#^*P* < 0.05, vs. the MCAO group. **(D)** Quantification analysis of H4K16ac. Values are expressed as mean ± SEM (*n* = 6). *P < 0.05, **P < 0.01, vs. the control group; ^**#**^*P* < 0.05, vs. the MCAO group. **(E)** Quantification analysis of Beclin1. Values are expressed as mean ± SEM (*n* = 6). *P < 0.05, vs. the control group; ^**#**^*P* < 0.05, vs. the MCAO group. **(F)** Quantification analysis of LC3-II values are expressed as mean ± SEM (*n* = 6). *P < 0.05, **P < 0.01, vs. the control group; ^#^*P* < 0.05, ^#^^#^*P* < 0.01, vs. the MCAO group.

To test the involvement of autophagy in CIR injury, we investigated the protein expression of LC3-II and Beclin1, which are effective markers for detection of autophagy (Figures 4E,F). The results revealed that the administration of EA enhanced the I/R-induced increases in the level of LC3-II and Beclin1, confirming that EA resulted in increased autophagic flux (Figures 4E,F). Cotreatment with hMOF siRNA, which inhibited hMOF to decrease the acetylation status of H4K16, led to further increases in LC3-II and Beclin1 expressions. In contrast, remarkable decreases were detected upon EA and Sirt1 inhibitor treatments. To provide evidence that decreased apoptosis by autophagy induction was associated with acetylation of H4K16, the protein expressions of hMOF, Sirt1, and H4K16ac were detected by western blot (Figures 4B–D). Obviously, the observed upregulation of hMOF and H4K16ac combined with the downregulation of Sirt1 upon I/R was abrogated when rats were treated with EA. Besides, inhibition of hMOF repressed the acetylation of H4K16, while Sirt1 siRNA reversed it. These data revealed that treatment with EA decreased the acetylation status of H4K16. Collectively, the results showed that I/R is related to H4K16ac-mediated autophagy and that maybe the mechanism of EA alleviates CIR injury.

### EA Effectively Regulates hMOF, Sirt1, and Beclin1 mRNA Expressions in the MCAO Rat Corpus Striatum

**Figure 5:**
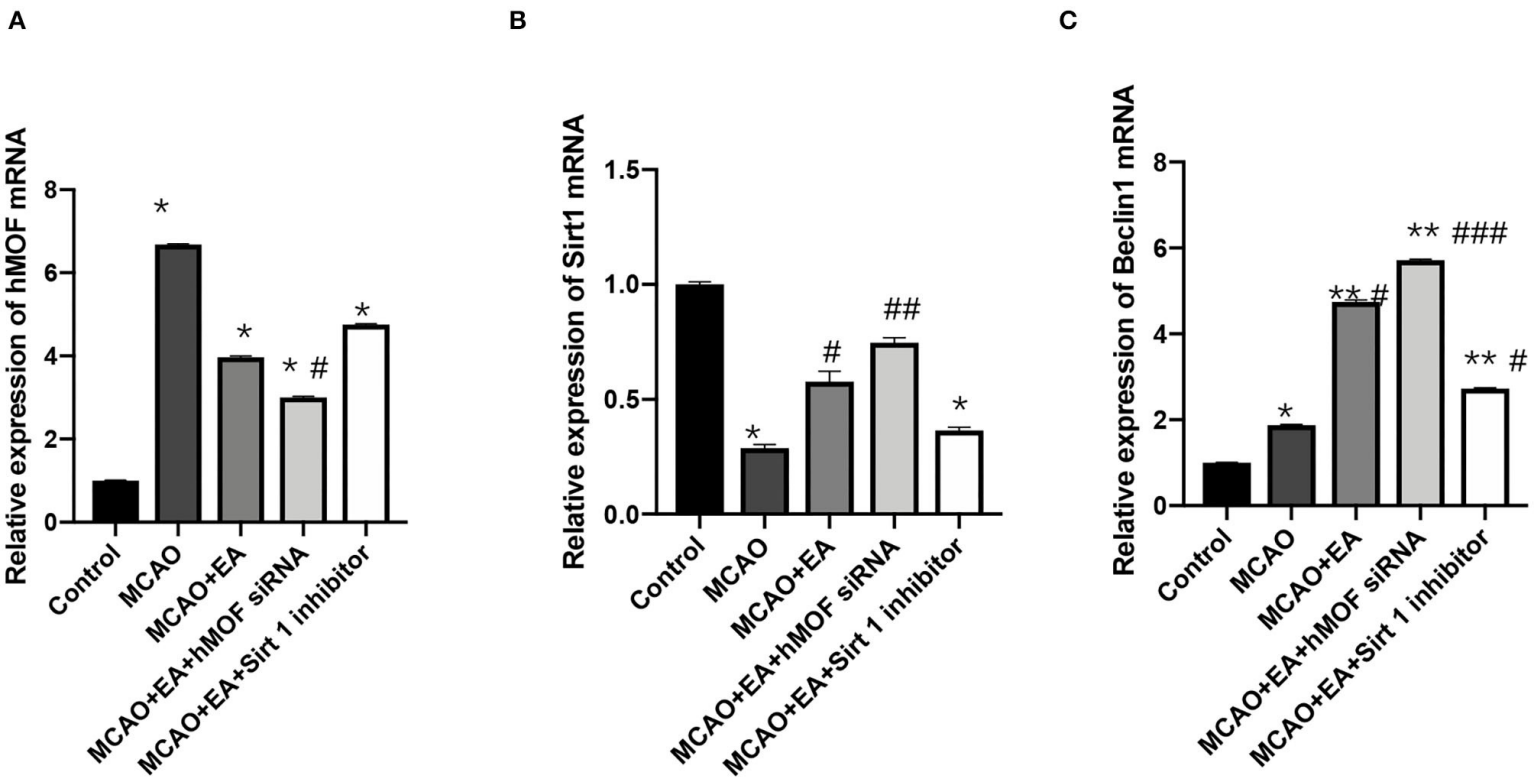
Electroacupuncture effectively regulated hMOF, Sirt1, and Beclin1 mRNA expressions in the MCAO rat corpus striatum. **(A)** Relative expression of hMOF mRNA. Values are expressed as mean ± SEM (*n* = 6). *P < 0.001, vs. the control group; ^**#**^*P* < 0.05, vs. the MCAO group. **(B)** Relative expression of Sirt1 mRNA. Values are expressed as mean ± SEM (*n* = 6). *P < 0.05, vs. the control group; ^**#**^*P* < 0.05, ^**##**^*P* < 0.01, vs. the MCAO group. **(C)** Relative expression of Beclin1 mRNA. Values are expressed as mean ± SEM (*n* = 6). *P < 0.01, **P < 0.001, vs. the control group; ^**#**^*P* < 0.05, ^**##**^*P* < 0.01, and ^**###**^*P* < 0.001, vs. the MCAO group.

qRT-PCR was performed to analyze the mRNA expressions of hMOF, Sirt1, and Beclin1 in the rat corpus striatum. Expression analysis demonstrated that treatment with I/R significantly increased hMOF and Beclin1 mRNA expressions (*P* < 0.05, Figures 5A,C). EA intervention enhanced the increase in Sirt1 and Beclin1 mRNA expressions (*P* < 0.05). A combination with hMOF siRNA led to similar results. However, there was no significant difference between the expression of hMOF mRNA in the MCAO+EA group compared with the MCAO group (*P* > 0.05). And EA cotreatment with the Sirt1 inhibitor inverted the tendency of the mRNAs of hMOF, Sirt1, and Beclin1, compared to EA treatment alone.

### EA Treatment Prominently Augments H4K16ac Occupancy

**Figure 6:**
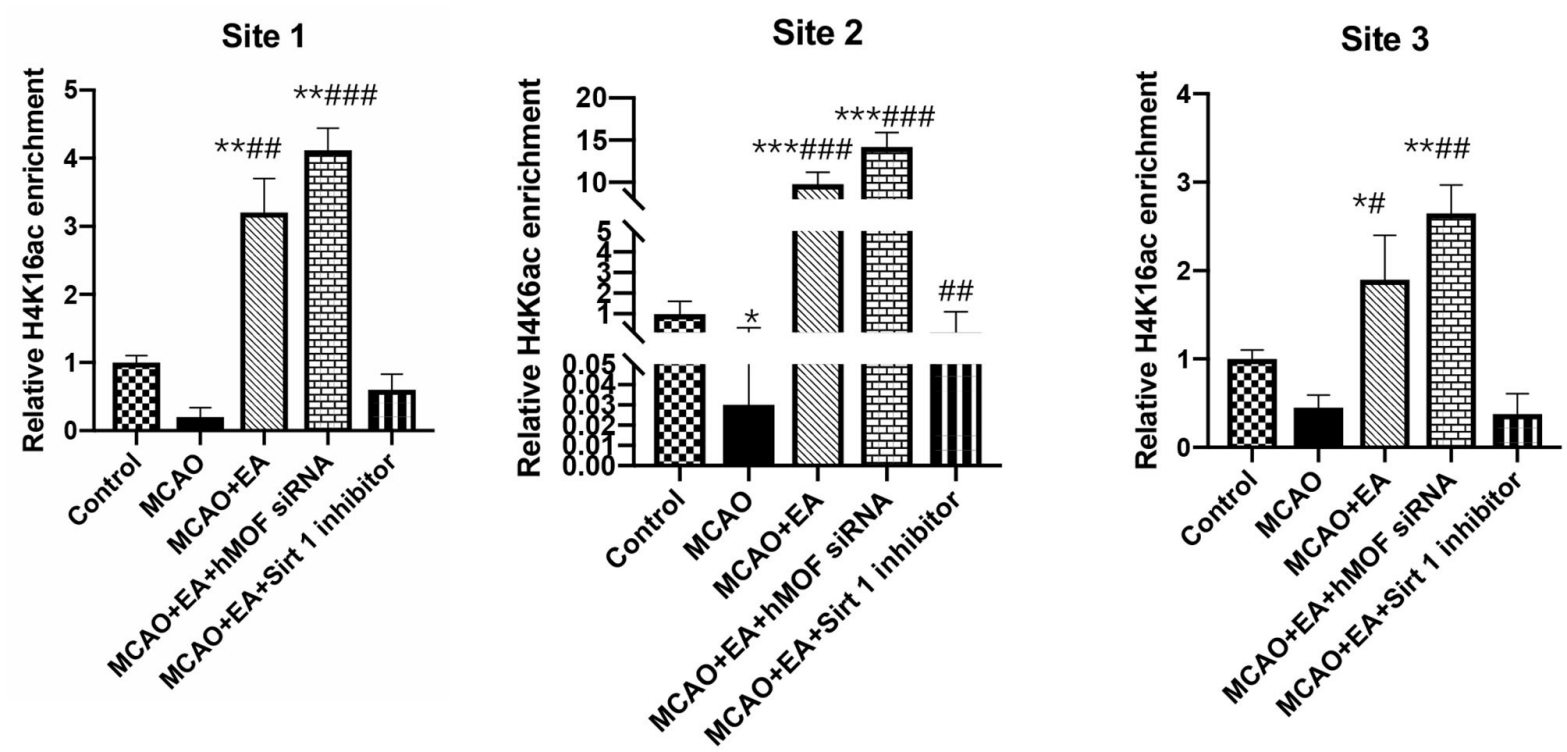
ChIP analysis of the enrichment of acetylated H4K16 on the Beclin1 promoter. ChIP quantitative analysis of extracted chromatin from the brains of each group and precipitated with H4K16ac antibody. Quantitative PCR was used to amplify the Beclin1 promoter region. The following formula was used to normalize the input results and nonspecific IgG results: [2 – (ΔCt) specific antibody/2 – (ΔCt) nonspecific IgG], where ΔCp is the Cp (immunoprecipitated DNA) – Cp(input) and Cp is the cycle where the threshold is crossed. The position at the top of each figure shows three different transcription positions of the Beclin1 promoter region. Data were expressed as means ± SD; *n* = 6, *P < 0.01, **P < 0.001, ***P < 0.0001, vs. the control group; ^**#**^*P* < 0.05, ^**##**^*P* < 0.01, and ^**###**^*P* < 0.001 vs. the MCAO group.

To investigate whether EA regulated autophagy-induced transcription and epigenetic regulation, we detected the binding of H4K16ac on the three sites in the Beclin1 promoter. Figure 6 shows the enrichment of H4K16ac chromatin on the three sites of the Beclin1 promoter. As shown, ChIP-qPCR assay increased the enrichment of the H4K16ac via EA treatment compared with the I/R condition (*P* < 0.01).

## Discussion

Ischemic stroke is one of the most ordinary and destructive stroke types, with high morbidity and mortality (27, 28). The occurrence and progress of ischemic stroke are caused by various factors (29). CIR injuries are often inevitable, while effective treatments for cerebral ischemia have not yet been discovered. Studies have confirmed that autophagy is involved in the regulation of various stages of cerebral ischemia (12–17). Autophagy refers to a catabolic process, which results in lysosomal degradation-dependent autophagy of large cytoplasmic contents and an excess of abnormal protein aggregates or damaged organelles. Autophagy is involved in various stages of cell growth, survival, and differentiation and has different assignments in anticancer, antiaging, antimicrobial defense, and neuroprotection. In summary, autophagy plays multiple roles in cerebral ischemic progression, but whether it is beneficial or pernicious remains unclear.

More than 30 autophagy-associated (ATG) proteins have been identified in yeast, most of which are conserved in mammals. In mammalian cells, the ATG protein is necessary for the correct execution of autophagy procedures. Beclin1 plays a vital role in the initiation of autophagy, and it forms a trimer with PI3K and Atg14 to recruit autophagy-related proteins to mediate the initiation of autophagy (30). As an indispensable regulator of autophagy, Beclin1 dominates the process of autophagy by regulating the production of class III PI3K-dependent phosphatidylinositol 3-phosphate (PI3P) and subsequent supplementation of ATG protein that promotes the formation of autophagosomes (31–33). In the past few years, much insight has been gained into how the function of Beclin1 scaffolds is regulated. It is essential for making clear the ability of Beclin1-dependent autophagy or non-autophagy to manipulate processes.

LC3 formation is derived from the precursor LC3 through proteolysis or deionization of autophagy LC3-PE. Under the action of cysteine protease Atg4, the LC3 precursor was processed into soluble LC3-I, which was related to the formation of lipid-soluble LC3-II-PE under phosphatidylethanolamine (PE) action, which was associated with the extension of the cell membrane of the body until the appearance of autophagosome (34, 35). LC3-II is the only reliable marker protein associated with complete autophagosomes and is also localized to phagosomes. Therefore, LC3-II is currently the primary biochemical marker of autophagy (36). In this paper, we used the relative level of Beclin1 and LC3-II to indicate the autophagic flux. In the MCAO group, the expression of LC3-II and Beclin1 showed a rising tendency compared to the control group insignificantly (*P* > 0.05). EA treatment facilitated the level of these two proteins ulteriorly. These results indicated that EA increased autophagic flux to alleviate I/R.

Literature shows that I/R-induced autophagic activation is coupled with the acetylation of H4K16 (37–40). Researchers noted that a molecular histone switch exists, where the balancing effects of hMOF and SIRT1 on H4K16 acetylation regulate autophagy (23). Studies have shown that H4K16 acetylation is linked to the nuclear deacetylase Sirt1 accumulation, which antagonizes the enzymatic activity of hMOF. This leads to hMOF deacetylation and activation of the promoter region of related genes (40). That is, the acetylation of H4K16 depends on the counteraction of hMOF and Sirt1 (41, 42).

Acupuncture for ischemic stroke is recommended by the World Health Organization (43, 44). Accumulating reports confirm that EA could alleviate I/R damage (9, 10). An underlying interrelation between the protective mechanism of acupuncture on ischemic stroke and autophagy has been found (11). For instance, Liu et al. (45) suggested that EA activated the mTORC1-ULK complex–Beclin1 pathway to inhibit autophagosome formation to induce autophagic flux to alleviate I/R injury. Wang et al. (46) showed that EA triggered Pink1/Parkin-mediated mitophagy clearance to ameliorate CIR-induced neuronal injury. Our previous studies demonstrated that EA inhibited the level of autophagy-related protein LC3-II, suppressed CIR injury, and regulated the expressions of histones H3K9 and H4K16 (47, 48). However, it remains elusive whether histone H4 lysine 16 acetylation plays a regulatory role in EA-induced suppression of I/R injury. Here, we showed that EA triggered the molecular histone switch of acetyltransferase (HATs) hMOF and histone deacetylase (HDACs) SIRT1 to reduce acetylation of H4K16 and facilitated MCAO-induced autophagy.

## Conclusion

To sum up, we hypothesize that regulating histone H4 lysine 16 acetylation-mediated autophagy may be a key mechanism of EA at Du20 and Du26 to treat I/R damage. EA treatment inhibited the H4K16ac process to facilitate autophagy and alleviated I/R injury in the end. However, there are some defects in this study as the lateral ventricular injection injury has not been excluded because of the limited number of groups, and we did not demonstrate this mechanism *in vitro*. Besides, the results showed that MCAO induced a small increase of autophagic flux, while EA treatment enhanced autophagy progress. These data indicated that the role of autophagy might vary with its level. The most appropriate autophagy flux to promote neurological function recovery should be focused on in the future.

## Data Availability Statement

All datasets generated for this study are included in the article/Supplementary Material.

## Ethics Statement

The animal study was reviewed and approved by the Animal Care and Use Committee of the First Affiliated Hospital of the Medical College at Nanjing traditional Chinese medicine University. Written informed consent was obtained from the owners for the participation of their animals in this study.

## Author Contributions

S-YX has made contribution to the conception and design of this work and taken part in the experiment studies. H-QL has done the experiment studies. W-QL has done data analysis and statistical analysis. HH has done the literature research. Y-JP and B-MZ have provided guidance throughout this study.

## Conflict of Interest

The authors declare that the research was conducted in the absence of any commercial or financial relationships that could be construed as a potential conflict of interest.
