# Supplementary figures and images for: Electroacupuncture Alleviates Cerebral Ischemia/Reperfusion Injury in Rats by Histone H4 Lysine 16 Acetylation-Mediated Autophagy
Source: Front Psychiatry. 2020 Dec 18;11:576539. doi: 10.3389/fpsyt.2020.576539 (PMC7775364; doi:10.3389/fpsyt.2020.576539)

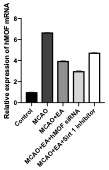

Supplement: Supplementary file 1 [file Data_Sheet_1.ZIP › biaoge/8.jpg]

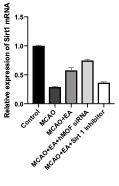

Supplement: Supplementary file 1 [file Data_Sheet_1.ZIP › biaoge/9.jpg]

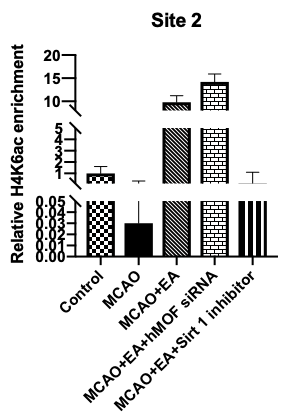

Supplement: Supplementary file 1 [file Data_Sheet_1.ZIP › biaoge/12.jpg]

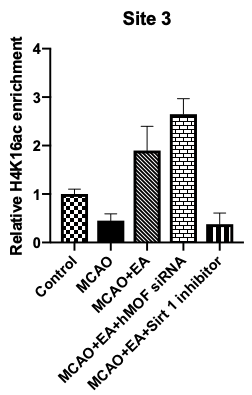

Supplement: Supplementary file 1 [file Data_Sheet_1.ZIP › biaoge/13.jpg]

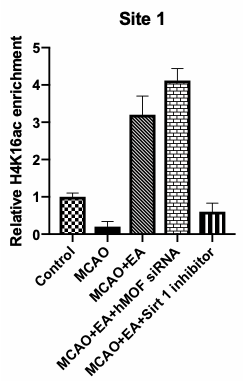

Supplement: Supplementary file 1 [file Data_Sheet_1.ZIP › biaoge/11.jpg]

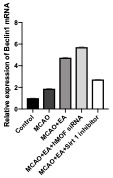

Supplement: Supplementary file 1 [file Data_Sheet_1.ZIP › biaoge/10.jpg]

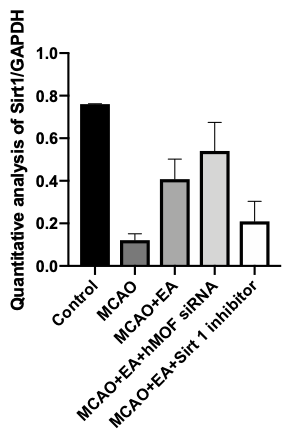

Supplement: Supplementary file 1 [file Data_Sheet_1.ZIP › biaoge/4.jpg]

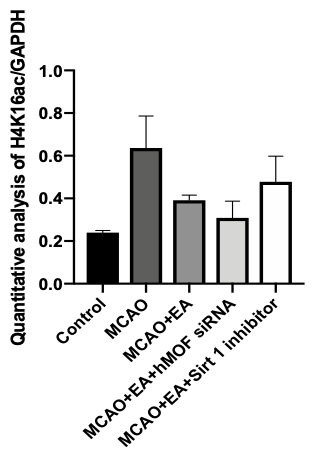

Supplement: Supplementary file 1 [file Data_Sheet_1.ZIP › biaoge/5.jpg]

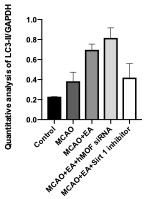

Supplement: Supplementary file 1 [file Data_Sheet_1.ZIP › biaoge/7.jpg]

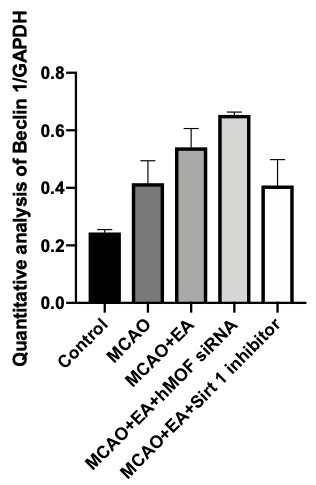

Supplement: Supplementary file 1 [file Data_Sheet_1.ZIP › biaoge/6.jpg]

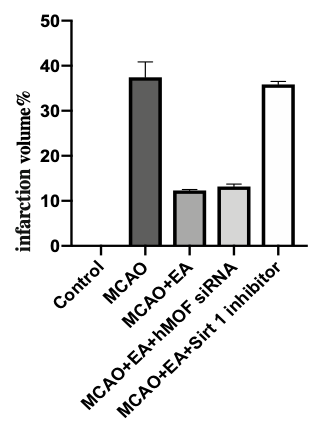

Supplement: Supplementary file 1 [file Data_Sheet_1.ZIP › biaoge/2.jpg]

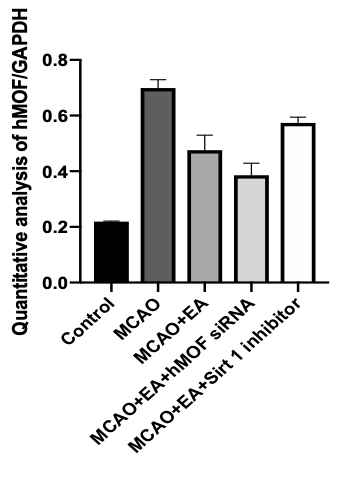

Supplement: Supplementary file 1 [file Data_Sheet_1.ZIP › biaoge/3.jpg]

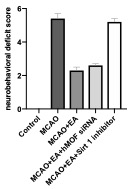

Supplement: Supplementary file 1 [file Data_Sheet_1.ZIP › biaoge/1.jpg]

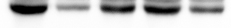

Supplement: Supplementary file 2 [file Data_Sheet_2.ZIP › τ1⁄41⁄46Θâ¿σêå∩╝ÜWBμúÇμ╡ï∩╝êτ╗äτ╗ç∩╝ë/Sirt1.jpg]

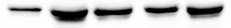

Supplement: Supplementary file 2 [file Data_Sheet_2.ZIP › τ1⁄41⁄46Θâ¿σêå∩╝ÜWBμúÇμ╡ï∩╝êτ╗äτ╗ç∩╝ë/hMOF.jpg]

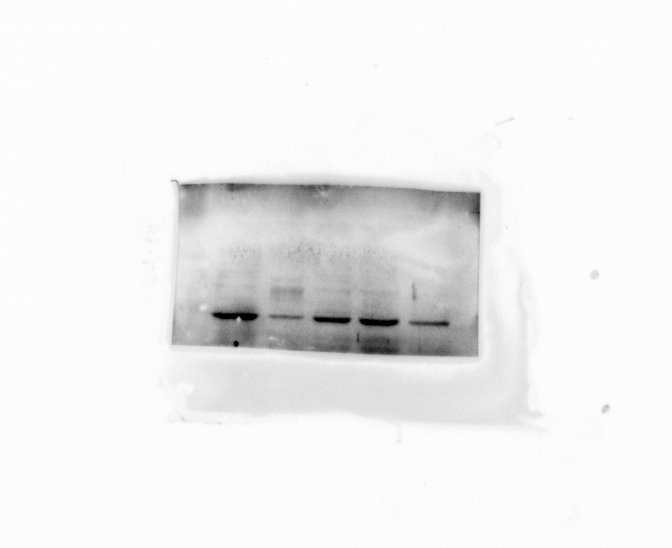

Supplement: Supplementary file 2 [file Data_Sheet_2.ZIP › τ1⁄41⁄46Θâ¿σêå∩╝ÜWBμúÇμ╡ï∩╝êτ╗äτ╗ç∩╝ë/H4K16ac.tif]

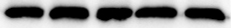

Supplement: Supplementary file 2 [file Data_Sheet_2.ZIP › τ1⁄41⁄46Θâ¿σêå∩╝ÜWBμúÇμ╡ï∩╝êτ╗äτ╗ç∩╝ë/GAPDH.jpg]

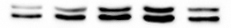

Supplement: Supplementary file 2 [file Data_Sheet_2.ZIP › τ1⁄41⁄46Θâ¿σêå∩╝ÜWBμúÇμ╡ï∩╝êτ╗äτ╗ç∩╝ë/LC3-II.jpg]

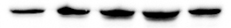

Supplement: Supplementary file 2 [file Data_Sheet_2.ZIP › τ1⁄41⁄46Θâ¿σêå∩╝ÜWBμúÇμ╡ï∩╝êτ╗äτ╗ç∩╝ë/Beclin-1.jpg]

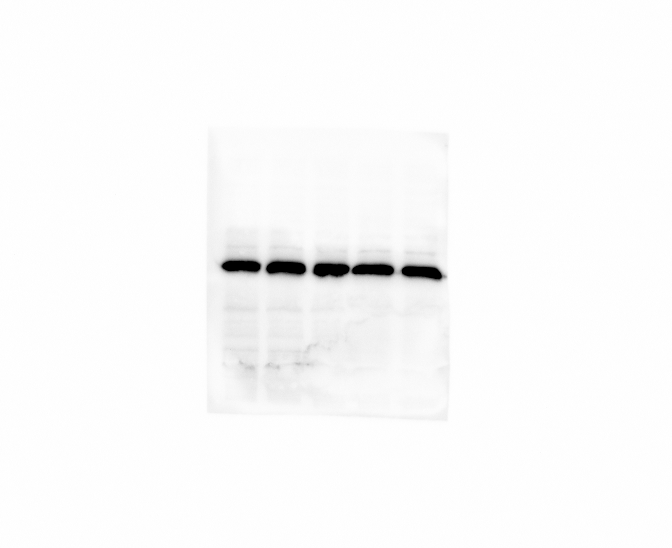

Supplement: Supplementary file 2 [file Data_Sheet_2.ZIP › τ1⁄41⁄46Θâ¿σêå∩╝ÜWBμúÇμ╡ï∩╝êτ╗äτ╗ç∩╝ë/GAPDH.tif]

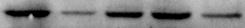

Supplement: Supplementary file 2 [file Data_Sheet_2.ZIP › τ1⁄41⁄46Θâ¿σêå∩╝ÜWBμúÇμ╡ï∩╝êτ╗äτ╗ç∩╝ë/H4K16ac.jpg]

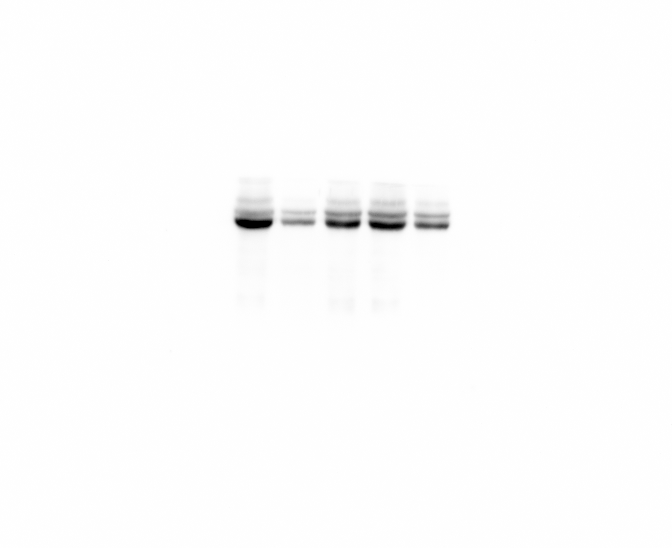

Supplement: Supplementary file 2 [file Data_Sheet_2.ZIP › τ1⁄41⁄46Θâ¿σêå∩╝ÜWBμúÇμ╡ï∩╝êτ╗äτ╗ç∩╝ë/Sirt1.tif]

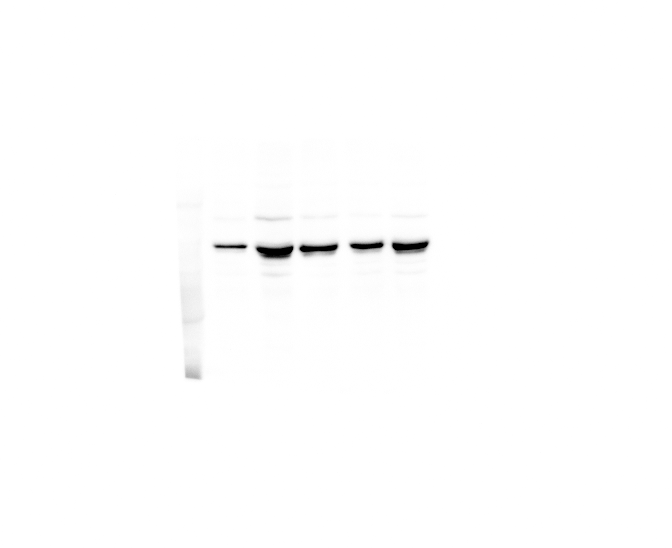

Supplement: Supplementary file 2 [file Data_Sheet_2.ZIP › τ1⁄41⁄46Θâ¿σêå∩╝ÜWBμúÇμ╡ï∩╝êτ╗äτ╗ç∩╝ë/hMOF.tif]

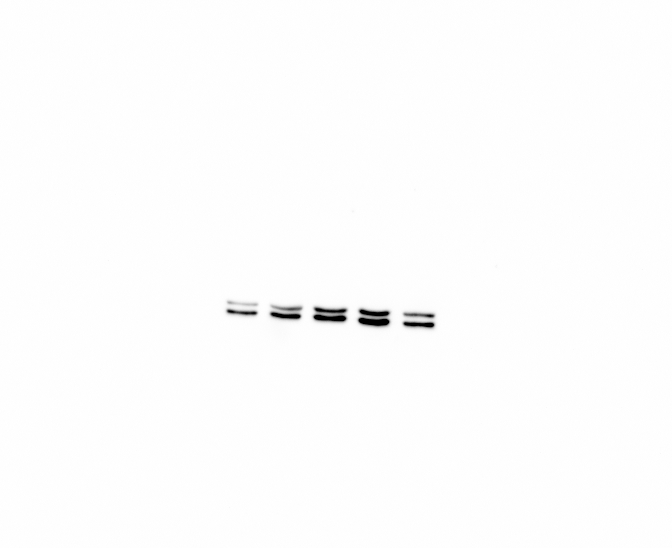

Supplement: Supplementary file 2 [file Data_Sheet_2.ZIP › τ1⁄41⁄46Θâ¿σêå∩╝ÜWBμúÇμ╡ï∩╝êτ╗äτ╗ç∩╝ë/LC3-II.tif]

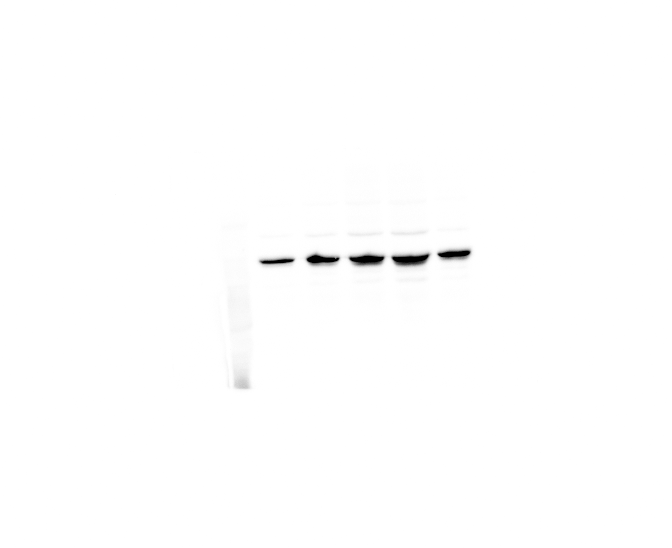

Supplement: Supplementary file 2 [file Data_Sheet_2.ZIP › τ1⁄41⁄46Θâ¿σêå∩╝ÜWBμúÇμ╡ï∩╝êτ╗äτ╗ç∩╝ë/Beclin-1.tif]
