# Supplementary material for: Electroacupuncture Alleviates Cerebral Ischemia/Reperfusion Injury in Rats by Histone H4 Lysine 16 Acetylation-Mediated Autophagy
Source: Front Psychiatry. 2020 Dec 18;11:576539. doi: 10.3389/fpsyt.2020.576539 (PMC7775364; doi:10.3389/fpsyt.2020.576539)
Supplement: Supplementary file 3 [file Table_1.DOCX]

**第一部分 大鼠MCAO模型构建、电针治疗及脑立体给药**


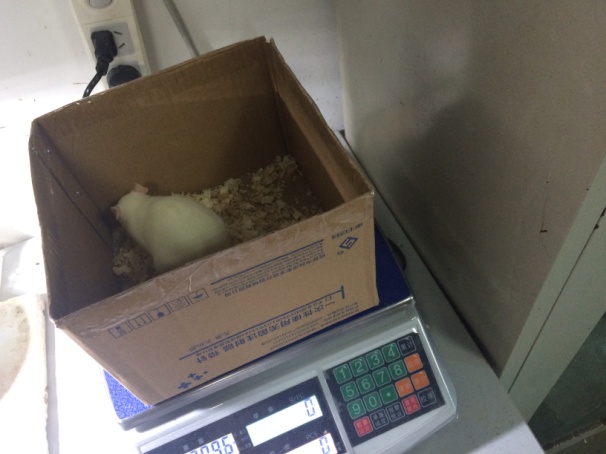

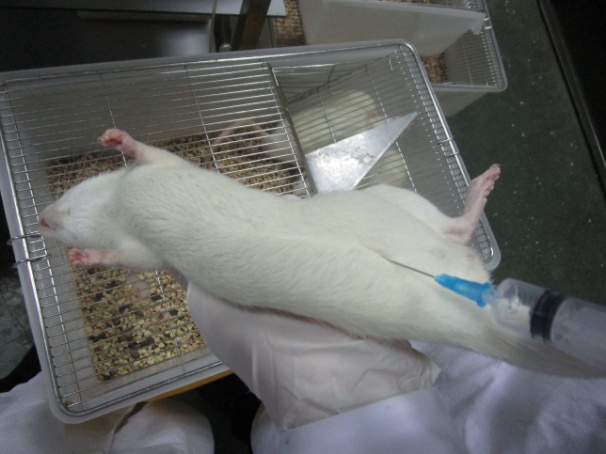


称重 麻醉


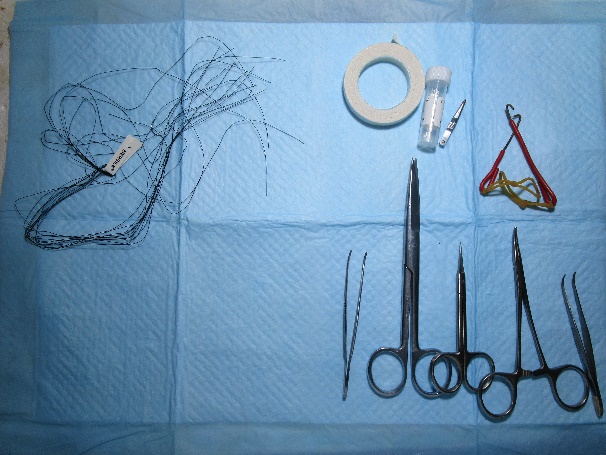

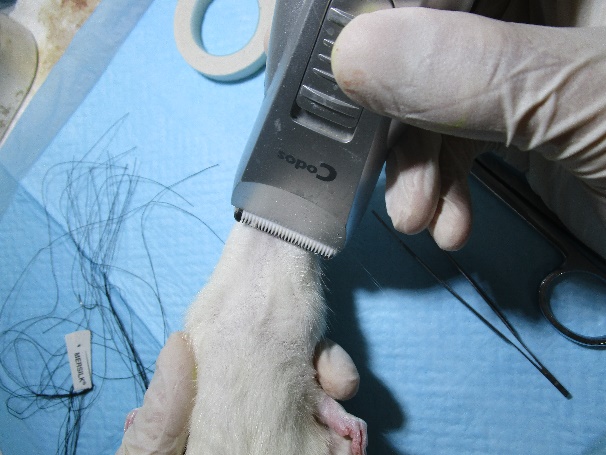


器械准备 备皮


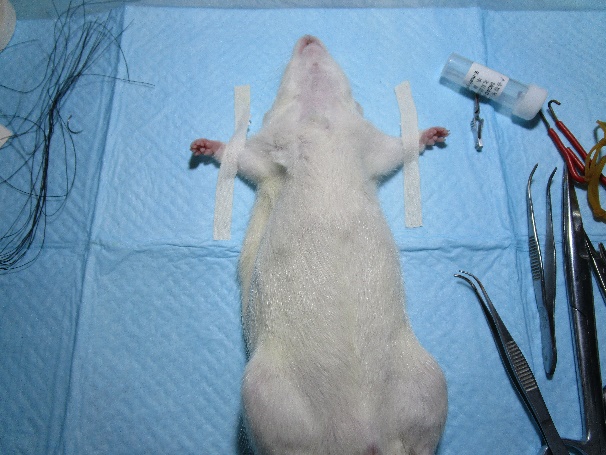

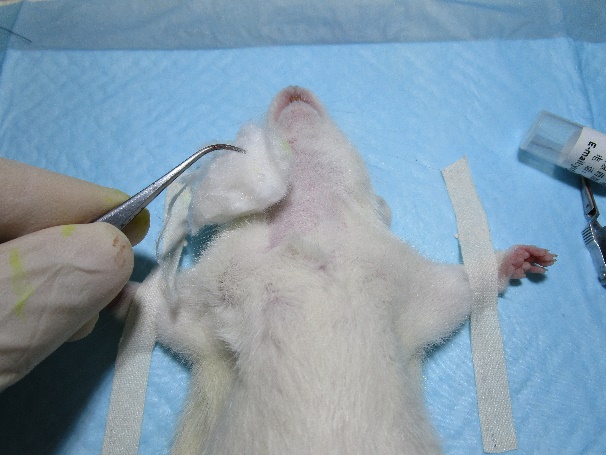


固定 消毒


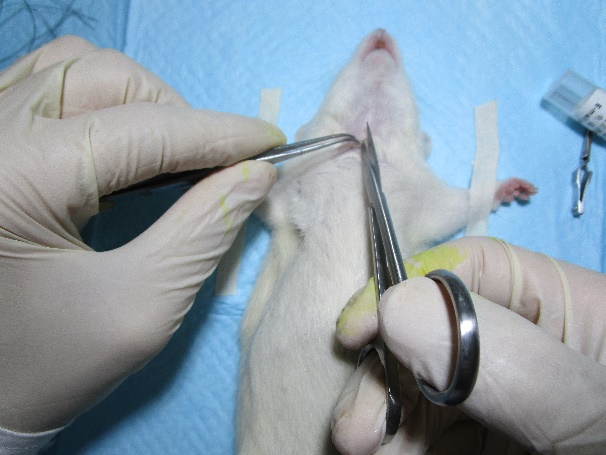

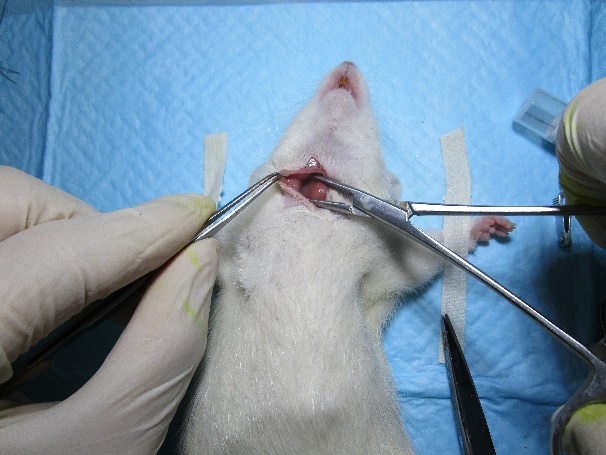


剪开外皮 钝性分离肌肉


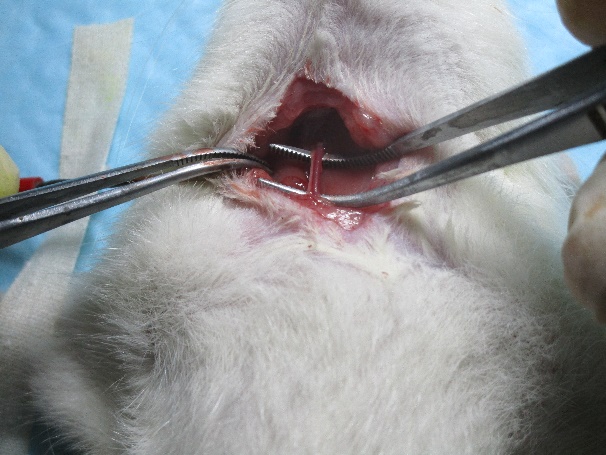

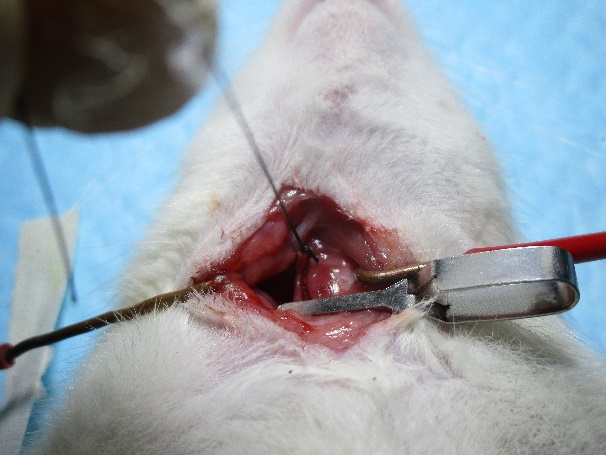


游离颈总动脉 结扎颈外动脉


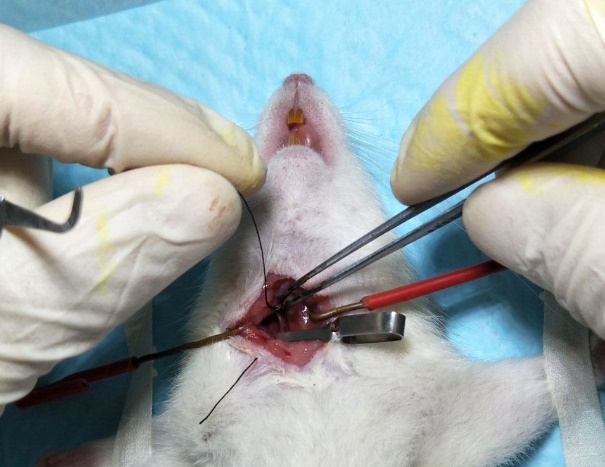

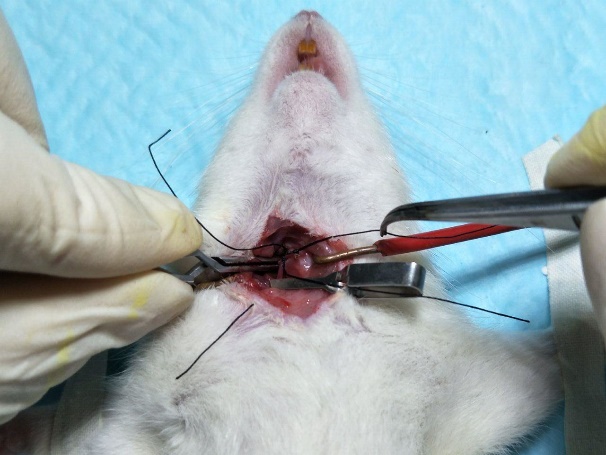


熔断颈外动脉 夹闭颈内动脉


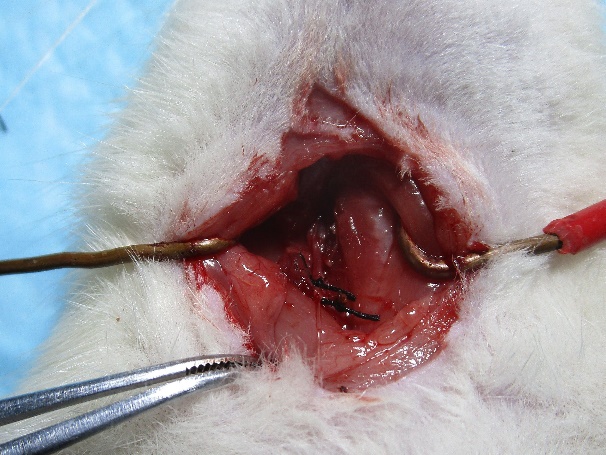

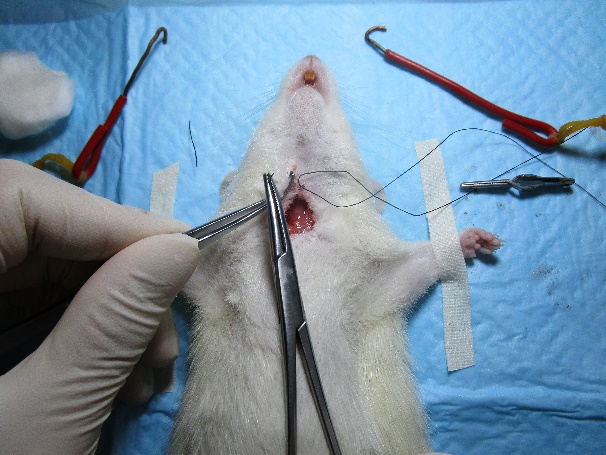


插入栓线后 缝合

**3.3 电针治疗**

电针时间点：第一次为造模成功后5min、第二次电针为第一次电针后16h。

穴位皮肤常规用75%酒精消毒后，用0.25*15mm华佗牌毫针，针刺大鼠的“人中（Du26）”和“百会（Du20）”穴。穴位定位参照《实验针灸学》（李忠仁主编，中国中医药出版社，2007：255）。“人中”穴在大鼠唇正中裂，鼻尖下1mm，0.5寸毫针向上斜刺1mm。“百汇”穴在头顶骨正中，平刺4mm。并给予电针治疗，接电针治疗仪，电针人中、百会，电流强度以大鼠胡须微微颤动为标准，采用疏密波，每次电针30分钟，共电针治疗两次。


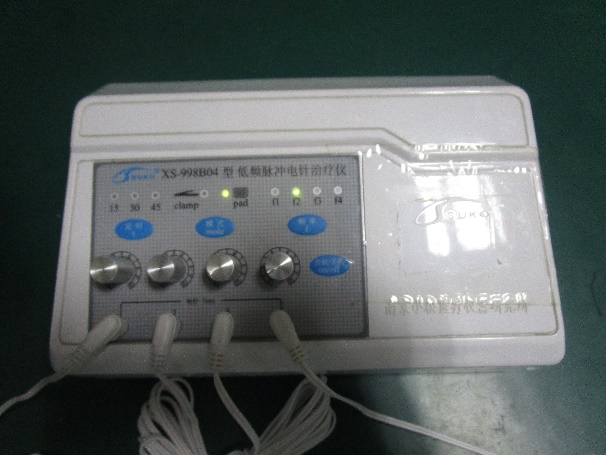

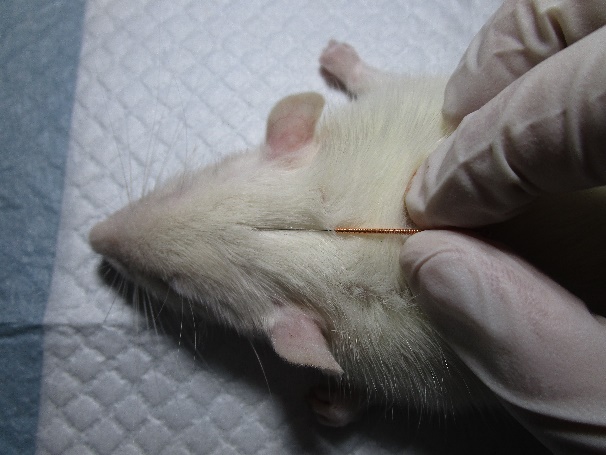


电针治疗仪 平刺百会穴


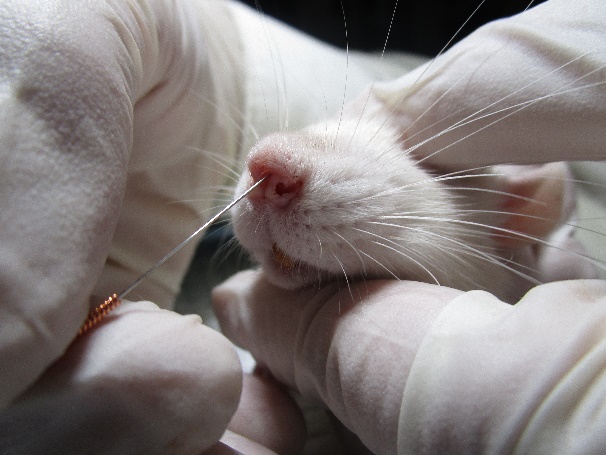

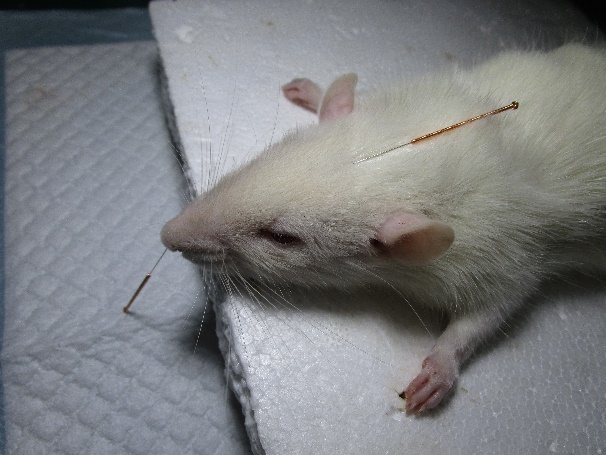


斜刺人中穴 针刺后


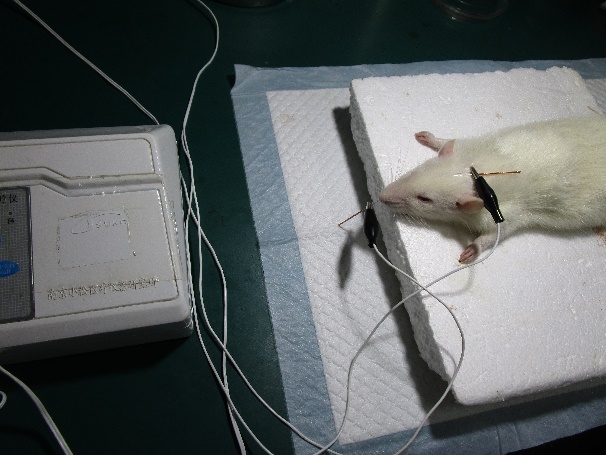


电针治疗

**3.4 侧脑室给药**


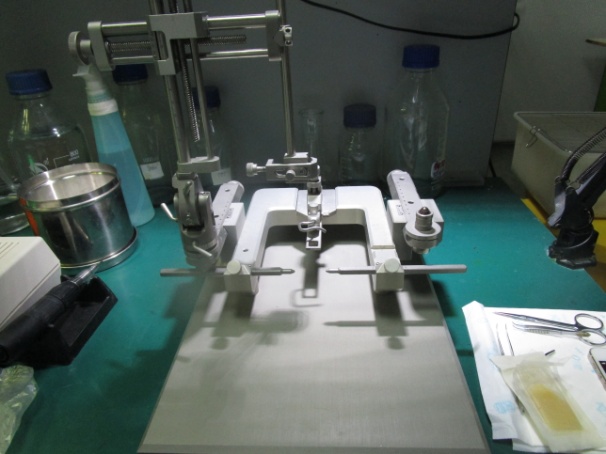

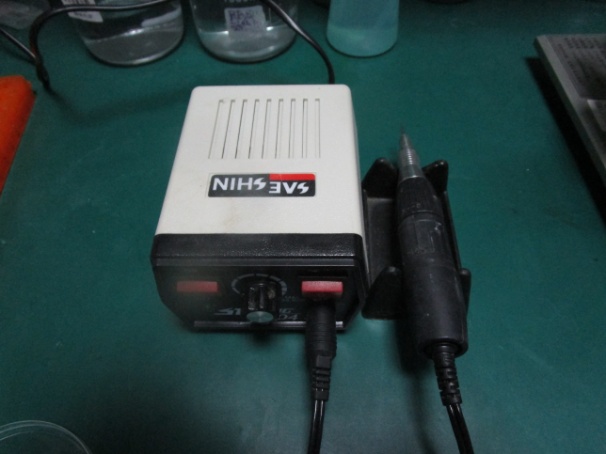


大鼠脑立体定位仪 高速颅骨钻


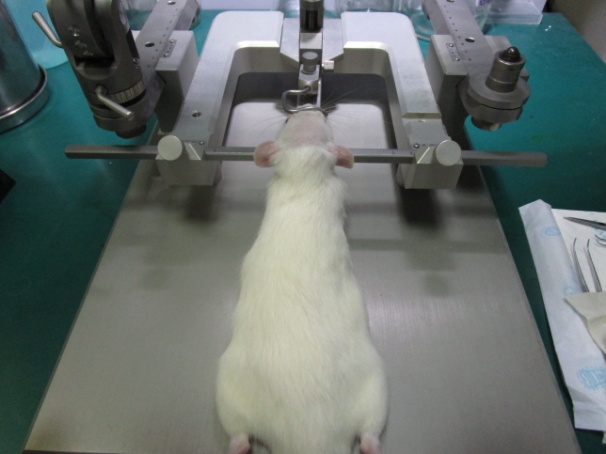

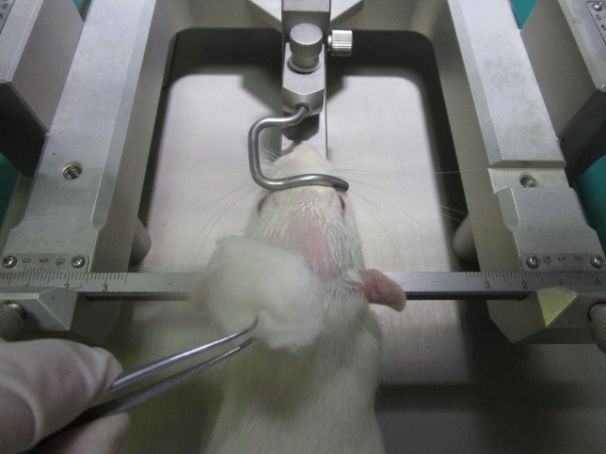


大鼠固定 消毒


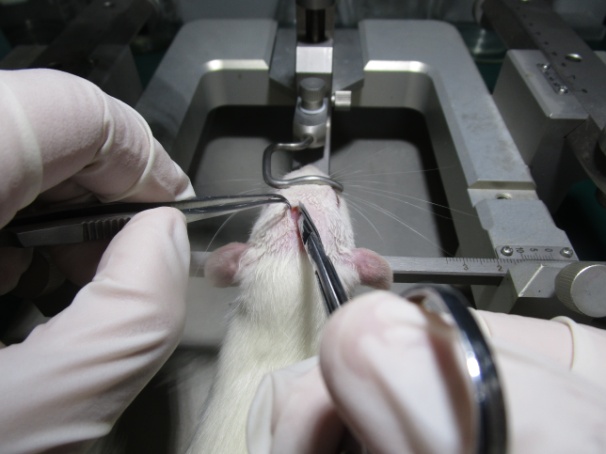

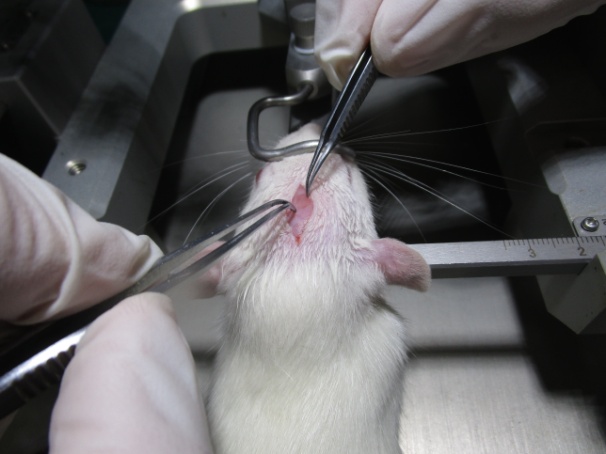


剪开头皮 剥离筋膜


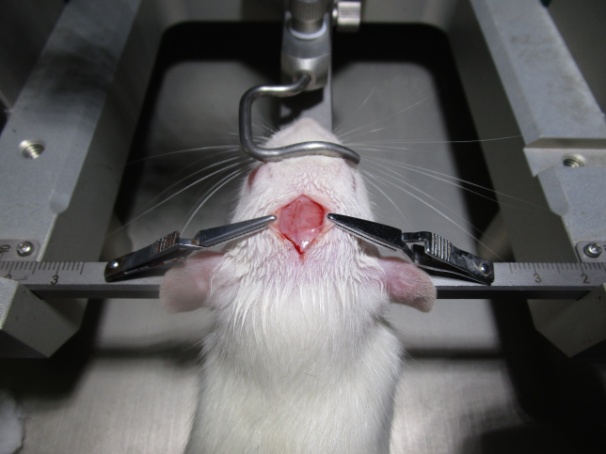

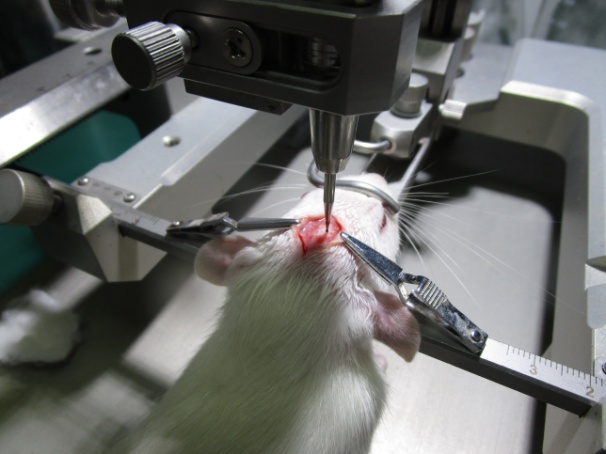


暴露视野 钻孔


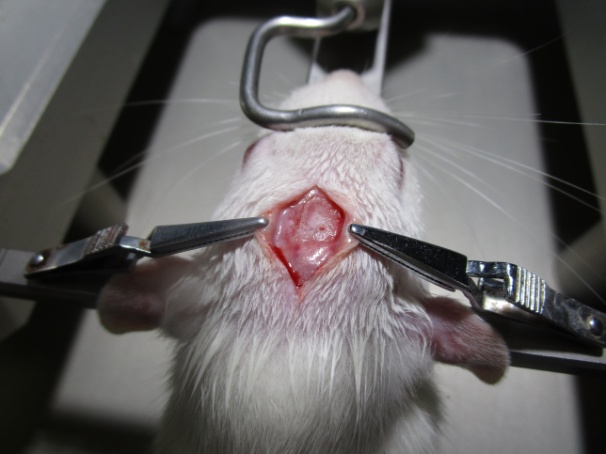

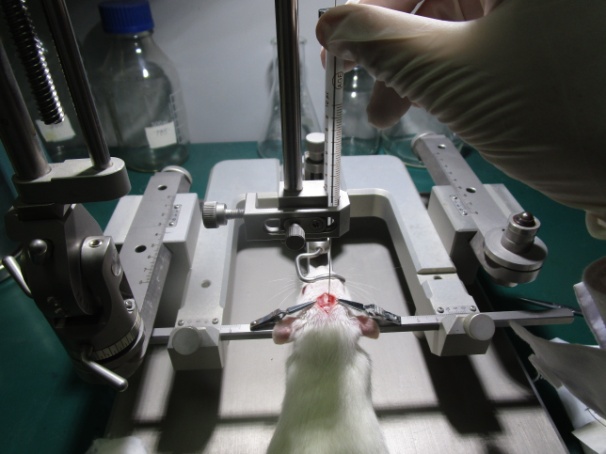


开孔后 注入药液


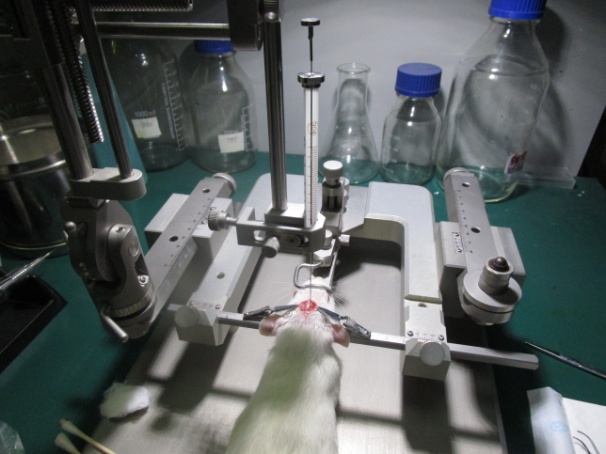

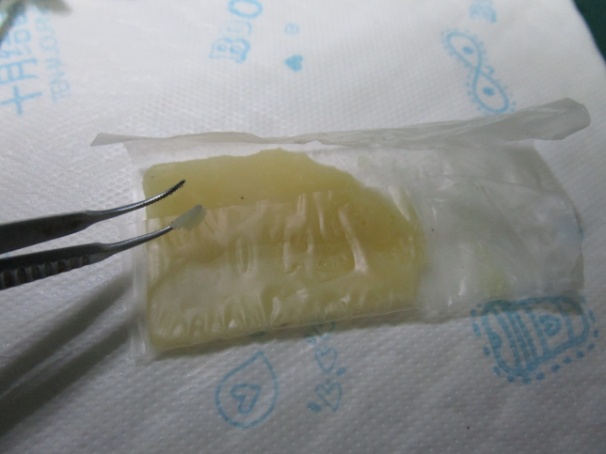


留针 骨蜡


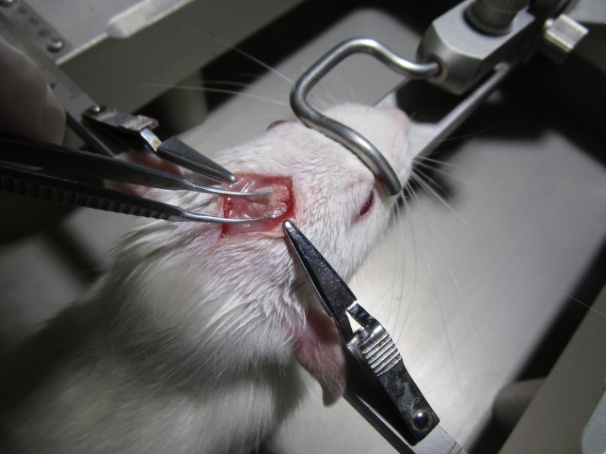

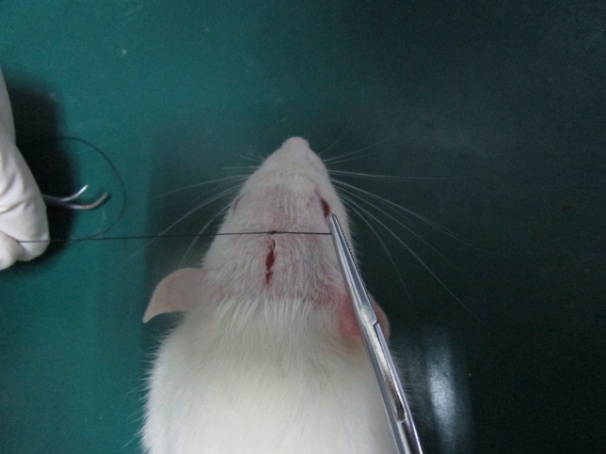


骨蜡封孔 缝合

**3.5 神经功能缺损评分**

第一次评分：筛选神经功能评分在1-3分的大鼠纳入实验对象，剔除神经功能评分为0分和4分的大鼠。

第二次评分：于取材前进行评分记录各组间大鼠神经功能评分的差异。

**评分表1：第一次造模处理后**

| 实验分组 | 动物编号 | 评分 | 动物编号 | 评分 |
| --- | --- | --- | --- | --- |
| A：正常对照组 | ① | 0 | ⑤ | 0 |
|  | ② | 0 | ⑥ | 0 |
|  | ③ | 0 | ⑦ | 0 |
|  | ④ | 0 |  |  |
| B：MACO模型组 |  | 3 | ⑥ | 3 |
|  |  | 4 | ⑦ | 3 |
|  |  | 5 | ⑧ | 死亡 |
|  |  | 6 | ⑨ | 3 |
|  |  | 3 |  |  |
| C：MACO模型+电针组 |  | 0 | ⑥ | 3 |
|  |  | 2 | ⑦ | 2 |
|  |  | 5 | ⑧ | 3 |
|  |  | 2 | ⑨ | 1 |
|  |  | 2 |  |  |
| D：MACO模型+电针+ hMOF siRNA干预组 |  | 2 | ⑥ | 2 |
|  |  | 2 | ⑦ | 1 |
|  |  | 4 | ⑧ | 0 |
|  |  | 3 | ⑨ | 死亡 |
|  |  | 2 |  |  |
| E：MACO模型+电针+ Sirt1抑制剂Nicotinamide组 |  | 2 | ⑥ | 2 |
|  |  | 6 | ⑦ | 3 |
|  |  | 4 | ⑧ | 4 |
|  |  | 2 | ⑨ | 3 |
|  |  | 2 |  |  |

**评分表2：取材前**

| 实验分组 | 动物编号 | 评分 | 动物编号 | 评分 |
| --- | --- | --- | --- | --- |
| A：正常对照组 | ① | 0 | ⑤ | 0 |
|  | ② | 0 | ⑥ | 0 |
|  | ③ | 0 | ⑦ | 0 |
|  | ④ | 0 |  |  |
| B：MACO模型组 | ① | 4 | ⑥ | 2 |
|  | ② | 2 | ⑦ | 3 |
|  | ③ | 3 | ⑧ |  |
|  | ④ | 3 | ⑨ |  |
|  | ⑤ | 3 |  |  |
| C：MACO模型+电针组 | ① |  | ⑥ | 2 |
|  | ② | 1 | ⑦ | 2 |
|  | ③ |  | ⑧ | 2 |
|  | ④ | 2 | ⑨ | 1 |
|  | ⑤ | 1 |  |  |
| D：MACO模型+电针+ hMOF siRNA干预组 | ① | 2 | ⑥ | 2 |
|  | ② | 1 | ⑦ | 2 |
|  | ③ | 2 | ⑧ |  |
|  | ④ | 3 | ⑨ |  |
|  | ⑤ | 2 |  |  |
| E：MACO模型+电针+ Sirt1抑制剂Nicotinamide组 | ① | 1 | ⑥ | 2 |
|  | ② | 2 | ⑦ | 1 |
|  | ③ | 2 | ⑧ |  |
|  | ④ | 3 | ⑨ |  |
|  | ⑤ | 2 |  |  |

**3.6 取材**

取材时间点：第二次侧脑室注射后7h。


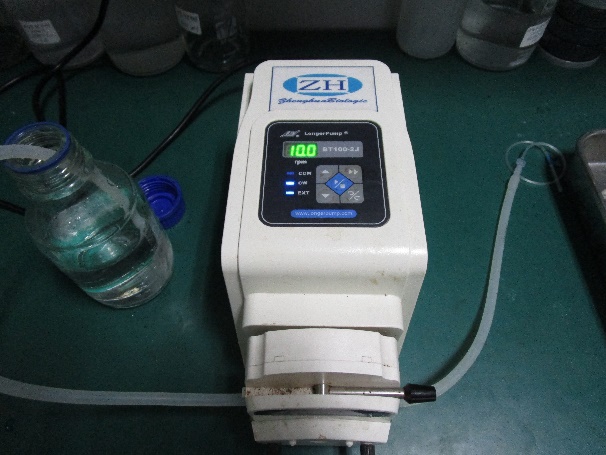

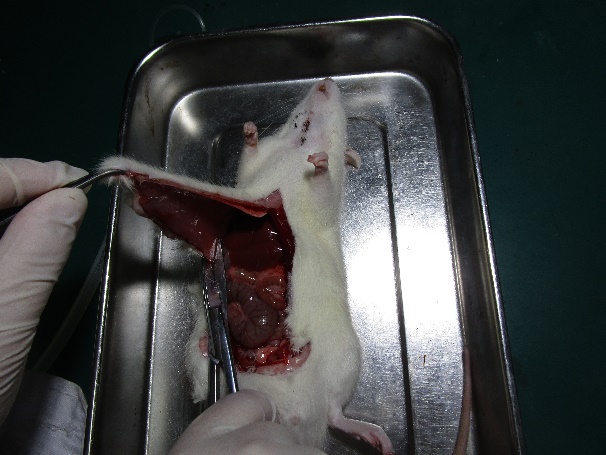


心脏灌流泵 剪开腹腔


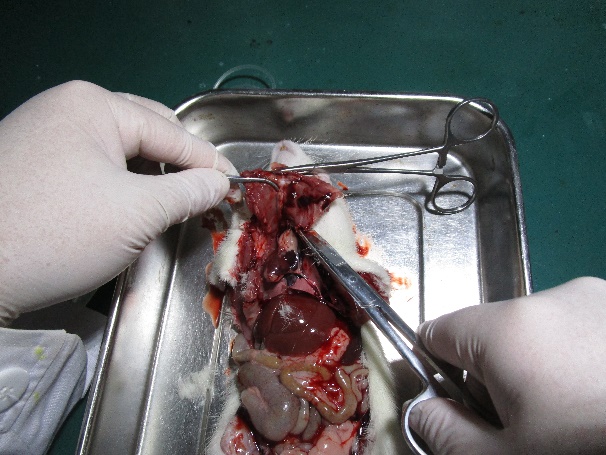

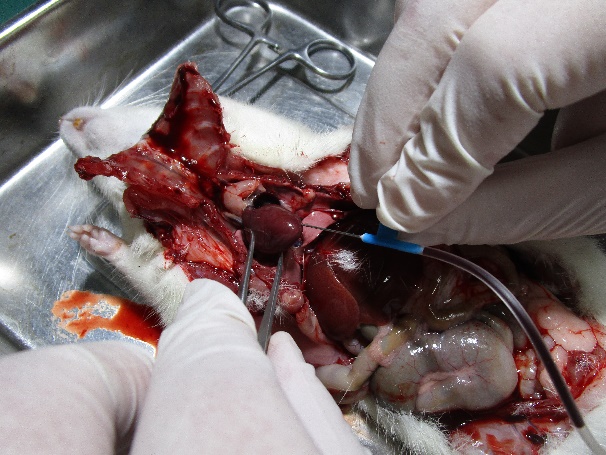


剪开胸腔 开始灌注


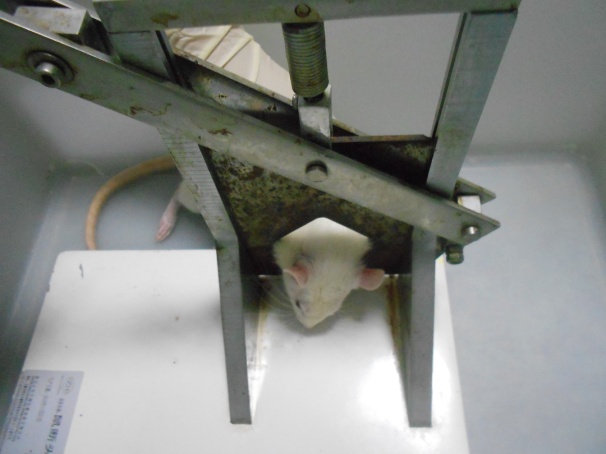

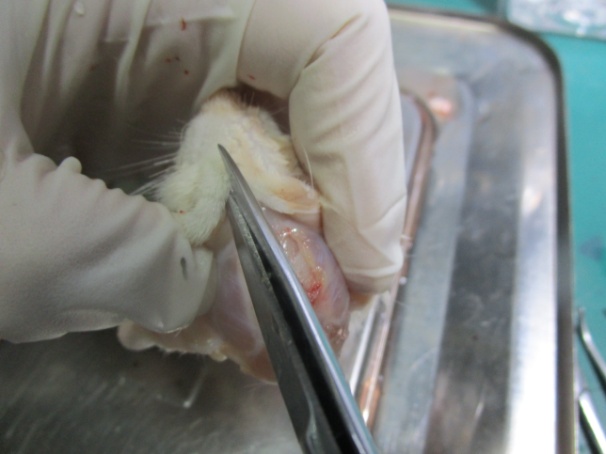


断头 剪开头皮


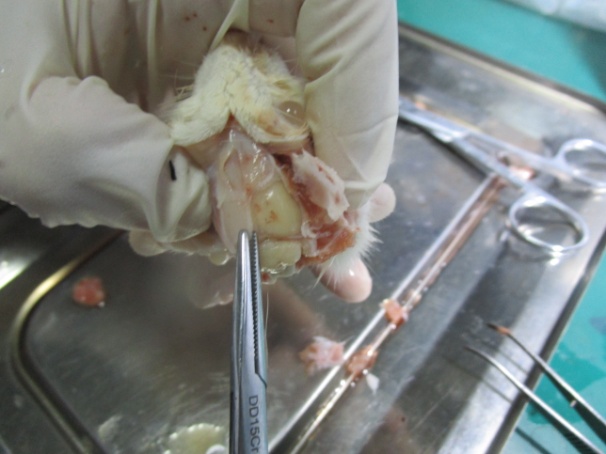

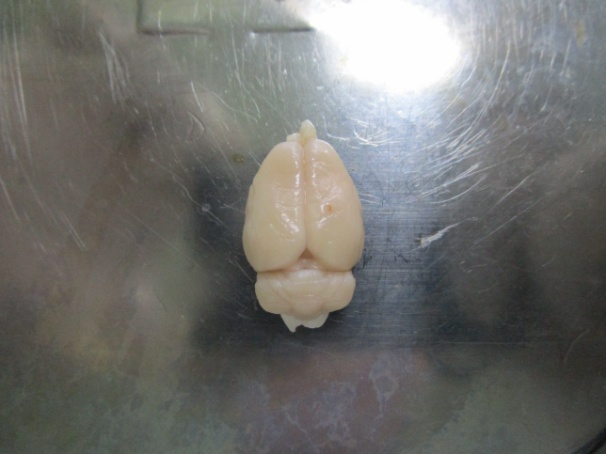


剥开颅骨 大脑


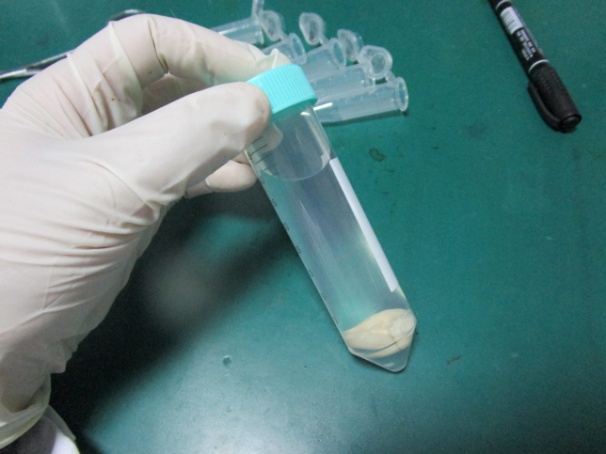
固定

**第二部分 TTC染色检测脑梗死灶分布**

1：正常对照组


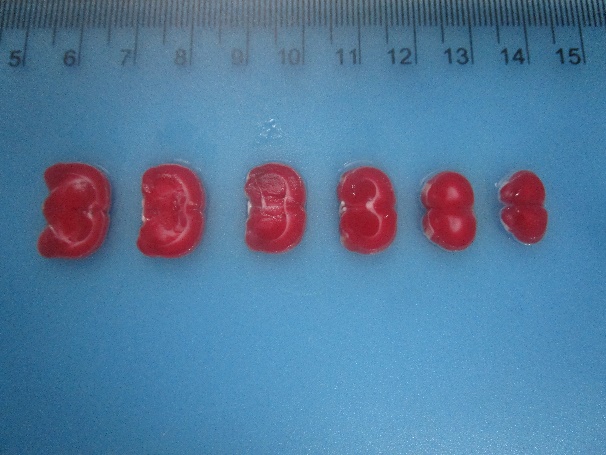

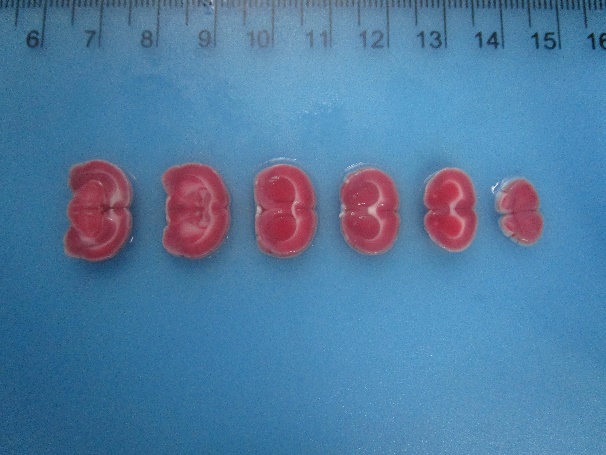


2：MCAO模型组


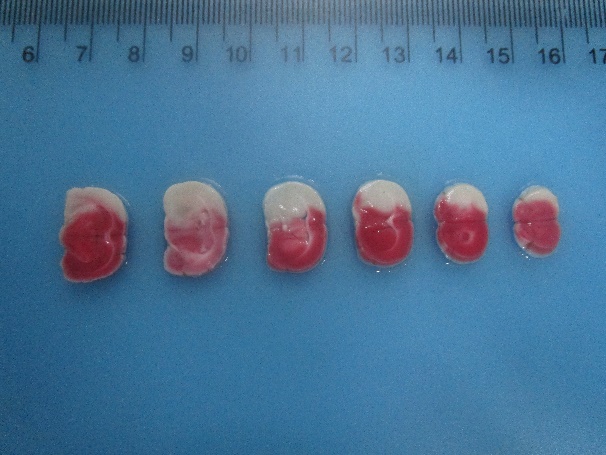

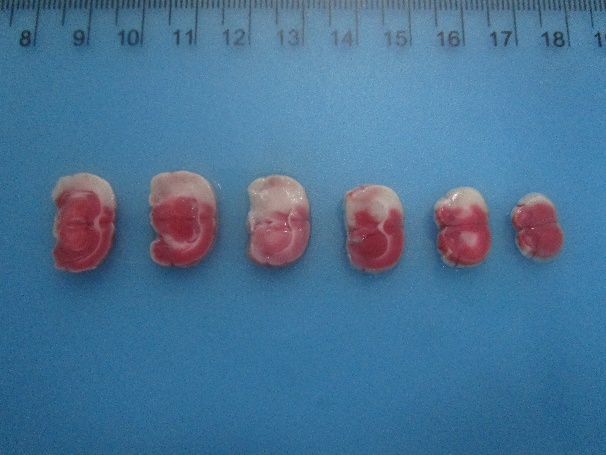


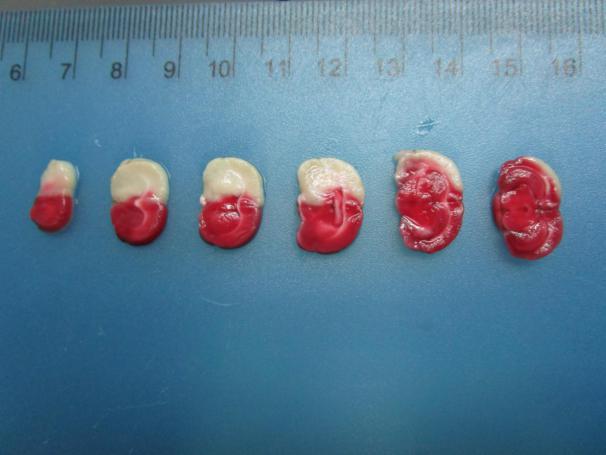

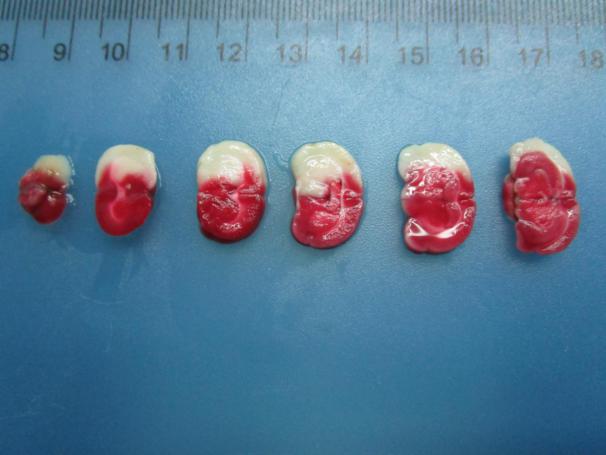


3：模型+电针组


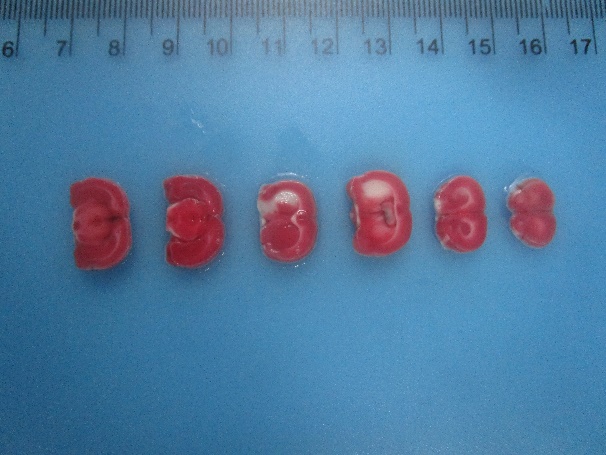

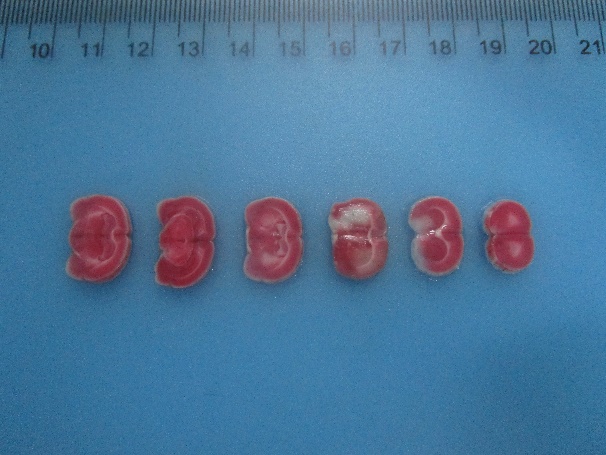


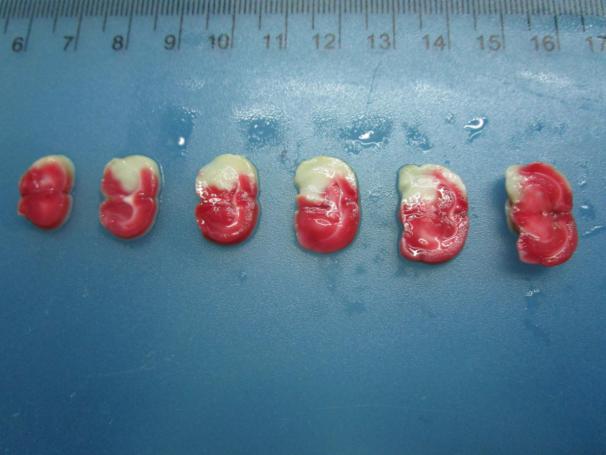

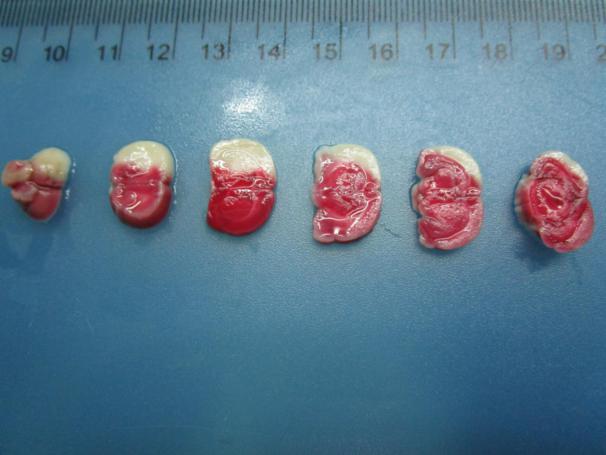


4：模型+电针组+hMOF siRNA干预组


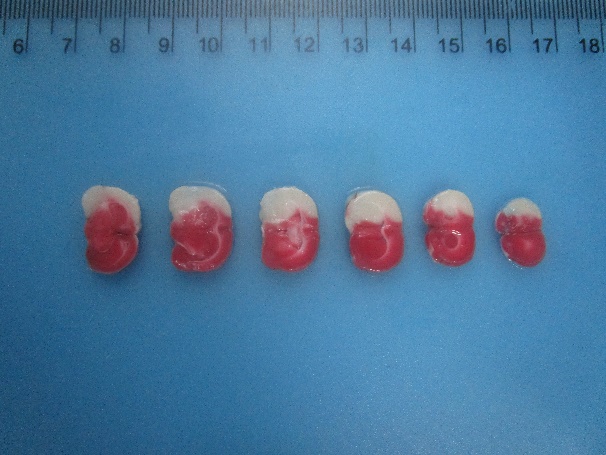

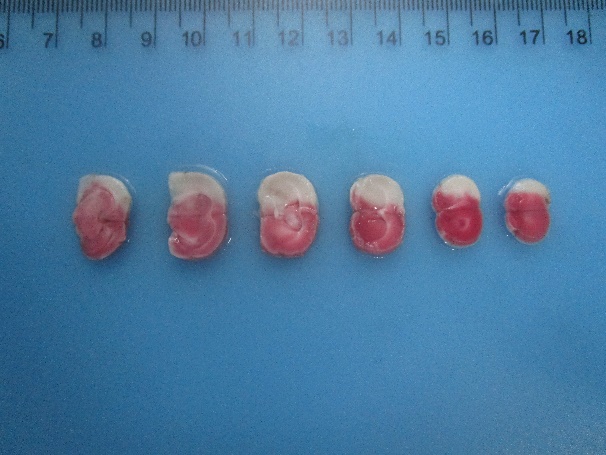


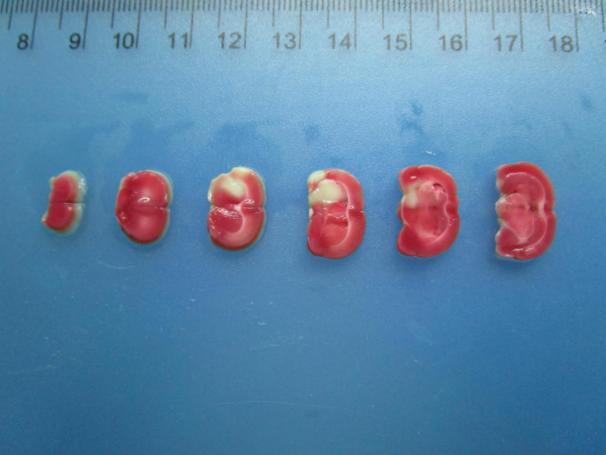

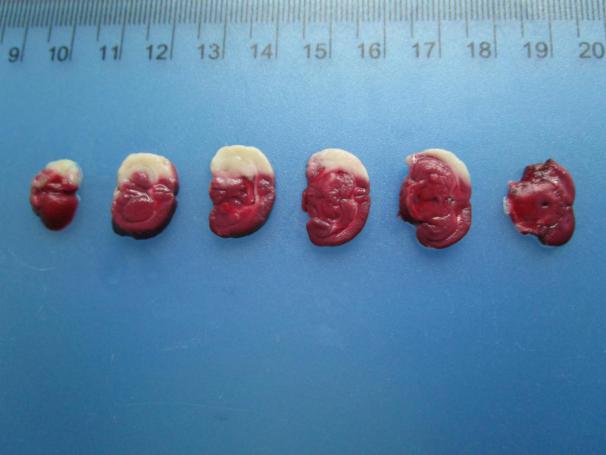


5：模型+电针组+Sirt1抑制剂组


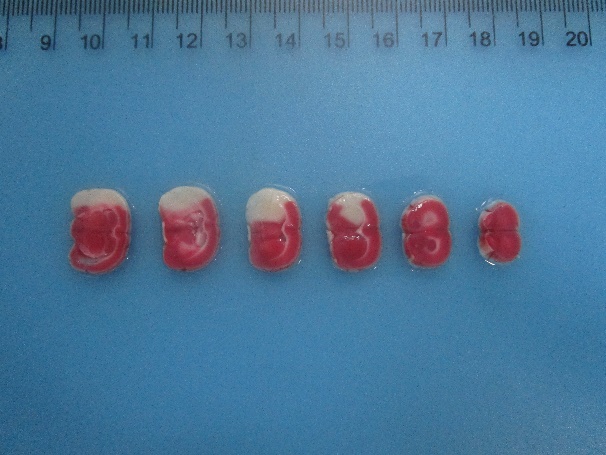

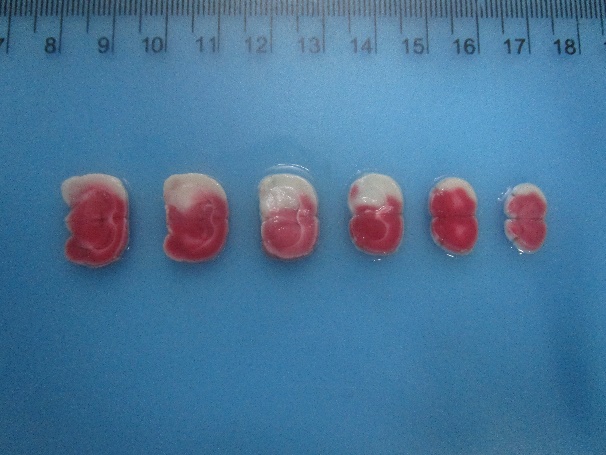


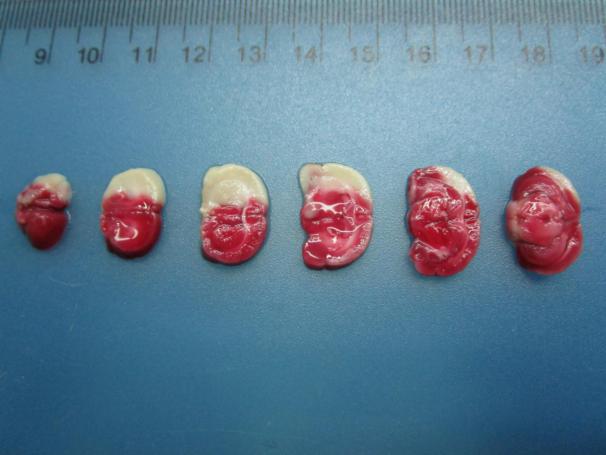

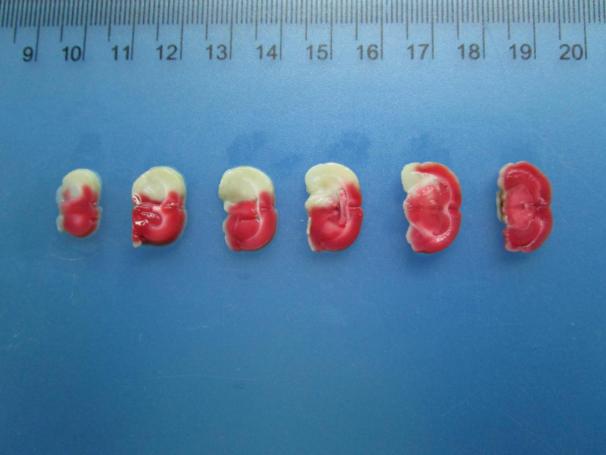


**第三部分 HE染色**

**5 实验结果**


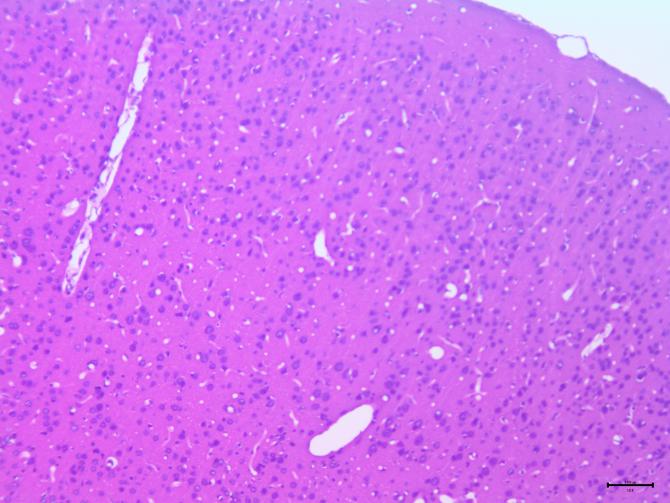

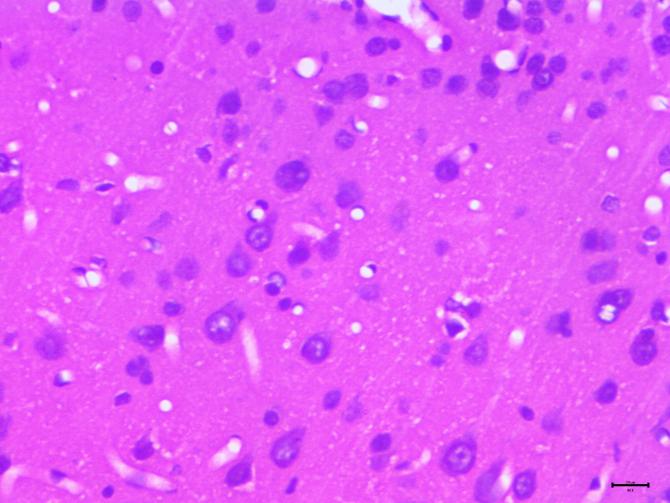


A-1-100X A-1-400X


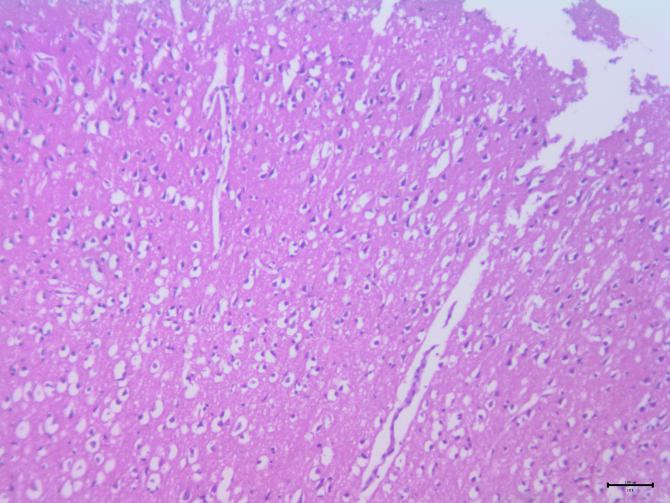

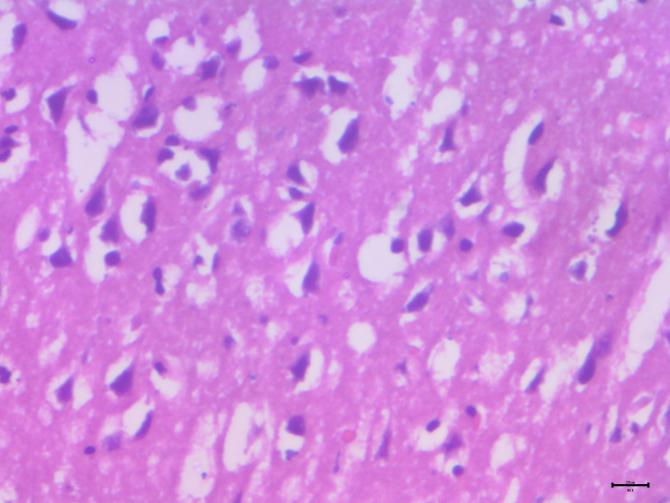


B-1-100X B-1-400X


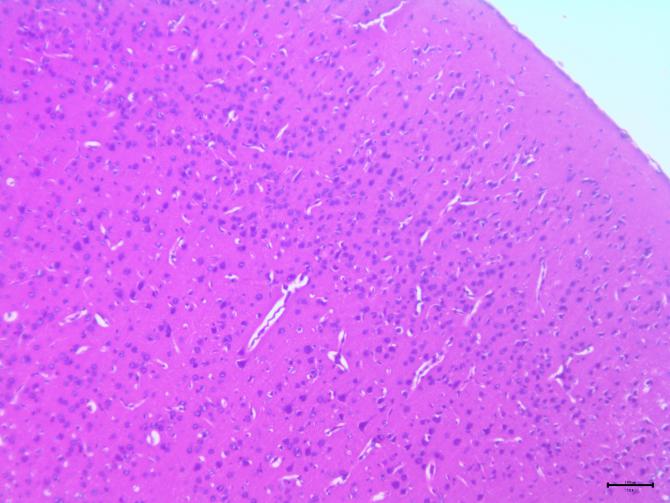

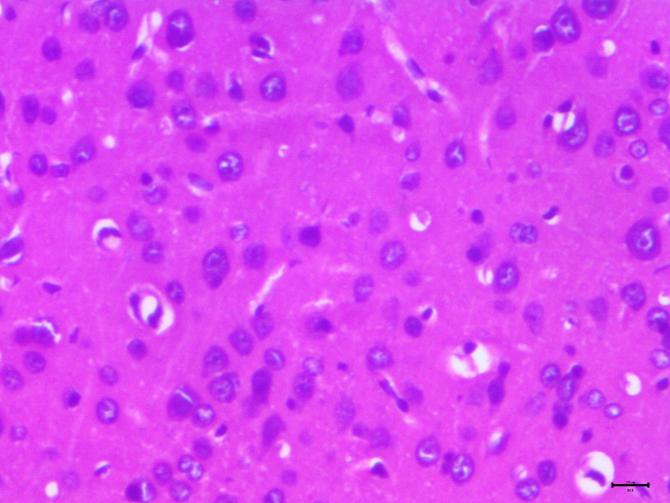


C-1-100X C-1-400X


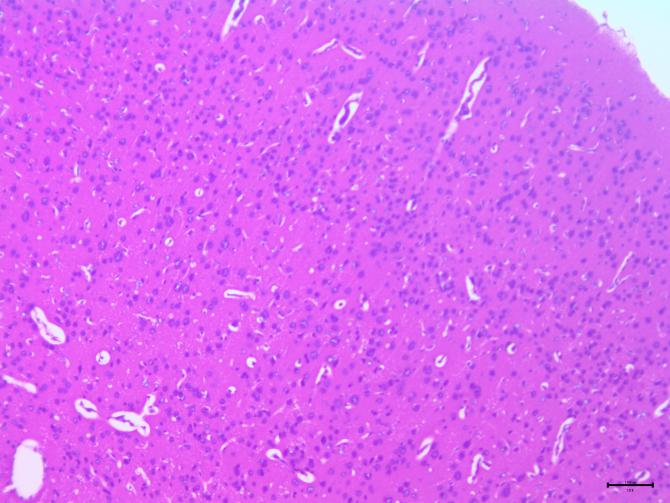

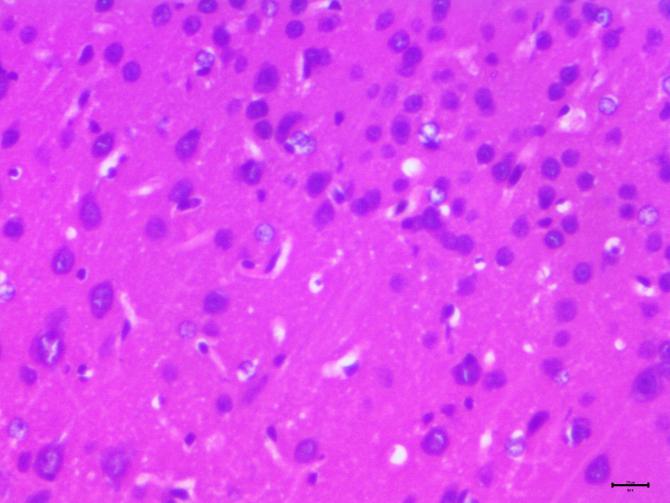


D-1-100X D-1-400X


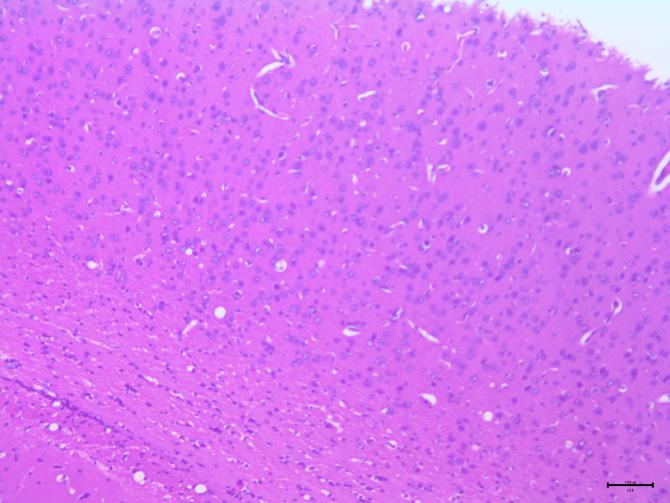

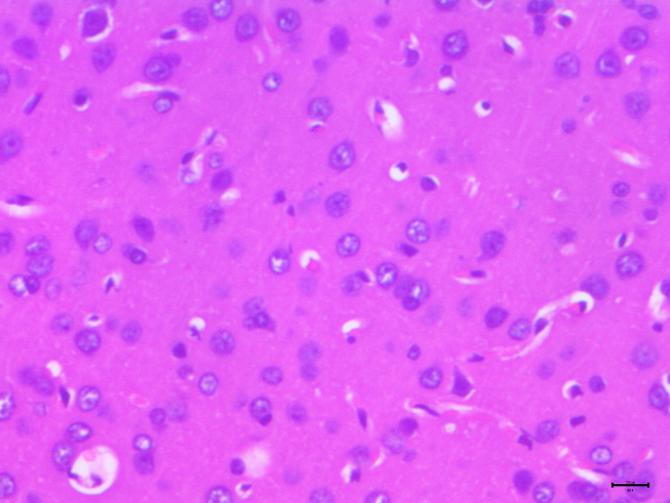


E-1-100X E-1-400X

1. **WB检测大鼠右侧纹状体脑组织中hMOF、Sirt1、H4K16ac、LC3-Ⅱ、Beclin1蛋白表达**

**5实验结果**

**5.1标准曲线及样品浓度**

| 标准品浓度（ug/mL） | OD1 | OD2 | 平均OD | 绝对OD |
| --- | --- | --- | --- | --- |
| 200 | 0.9982 | 0.9939 | 0.9961 | 0.9517 |
| 150 | 0.7898 | 0.7986 | 0.7942 | 0.7498 |
| 100 | 0.6016 | 0.6074 | 0.6045 | 0.5601 |
| 50 | 0.3975 | 0.3845 | 0.3910 | 0.3466 |
| 20 | 0.2416 | 0.2453 | 0.2435 | 0.1991 |
| 10 | 0.1616 | 0.1653 | 0.1635 | 0.1191 |
| 5 | 0.0963 | 0.0986 | 0.0975 | 0.0531 |
| 0 | 0.0438 | 0.0450 | 0.0444 | 0.0000 |


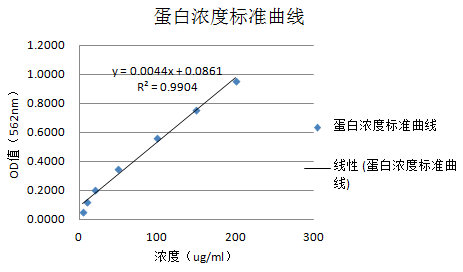


样本计算浓度为：

| 样本 | OD1 | OD2 | OD3 | 平均 | 样本终浓度（ug/ml） | 上样量 |
| --- | --- | --- | --- | --- | --- | --- |
| 1 | 0.6803 | 0.6855 | 0.6778 | 0.6812 | 12515.9091 | 20ug |
| 2 | 0.7291 | 0.7122 | 0.7106 | 0.7173 | 13336.3636 | 20ug |
| 3 | 0.6680 | 0.6755 | 0.6833 | 0.6756 | 12388.6364 | 20ug |
| 4 | 0.5982 | 0.5984 | 0.5967 | 0.5978 | 10619.6970 | 20ug |
| 5 | 0.7855 | 0.7865 | 0.7878 | 0.7866 | 14911.3636 | 20ug |

**5.2样本WB结果**

解析：蛋白条带灰度值用于衡量蛋白表达的多少，经内参校正后，灰度值越高，表明该目标蛋白表达越多。（仅供参考）

1 2 3 4 5


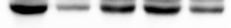
Sirt1；110KD


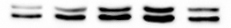
LC3-II；14，16KD


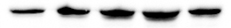
Beclin1；52KD


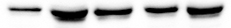
 hMOF；52KD


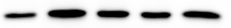
 H4K16ac；11KD


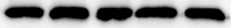
 GAPDH；36KD

**灰度分析：**

| 编号 | 1 | 2 | 3 | 4 | 5 |
| --- | --- | --- | --- | --- | --- |
| Sirt1 | 76.01 | 12.73 | 41.26 | 55.79 | 21.34 |
| LC3-Ⅱ | 22.85 | 40.29 | 70.45 | 84.19 | 42.65 |
| Beclin1 | 24.49 | 43.85 | 54.68 | 67.47 | 41.55 |
| hMOF | 21.87 | 73.61 | 48.06 | 39.82 | 58.34 |
| H4K16ac | 23.94 | 66.98 | 39.53 | 31.86 | 48.65 |
| GAPDH | 100.00 | 105.26 | 101.05 | 103.16 | 101.71 |

内参校正后：

| 编号 | 1 | 2 | 3 | 4 | 5 |
| --- | --- | --- | --- | --- | --- |
| Sirt1 | 76.01 | 12.09 | 40.83 | 54.08 | 20.98 |
| LC3-Ⅱ | 22.85 | 38.28 | 69.72 | 81.61 | 41.93 |
| Beclin1 | 24.49 | 41.66 | 54.11 | 65.40 | 40.85 |
| hMOF | 21.87 | 69.93 | 47.56 | 38.60 | 57.36 |
| H4K16ac | 23.94 | 63.63 | 39.12 | 30.88 | 47.83 |
| GAPDH | 100.00 | 100.00 | 100.00 | 100.00 | 100.00 |


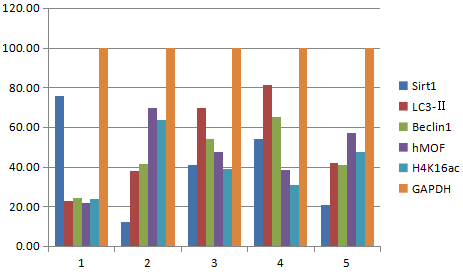


**5.3 实验操作图片**


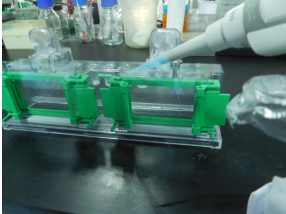

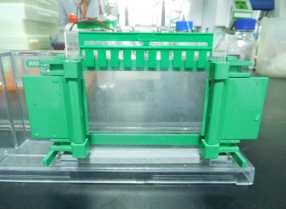


制胶 制胶


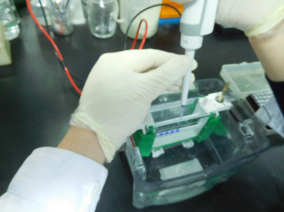

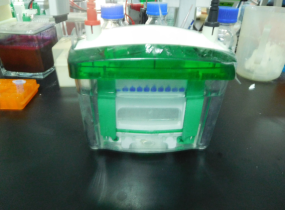


加样 跑胶


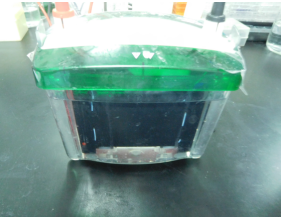

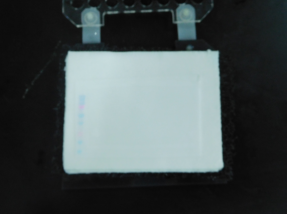


转膜 转膜完成


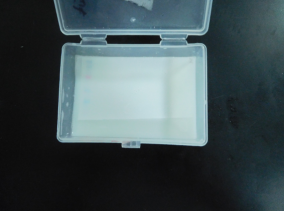

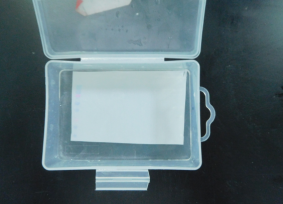


封闭 抗体孵育

**第七部分 QPCR检测大鼠右侧纹状体脑组织中hMOF、Sirt1、Beclin1mRNA表达**

**7实验数据结果统计**

7.1 QPCR视图


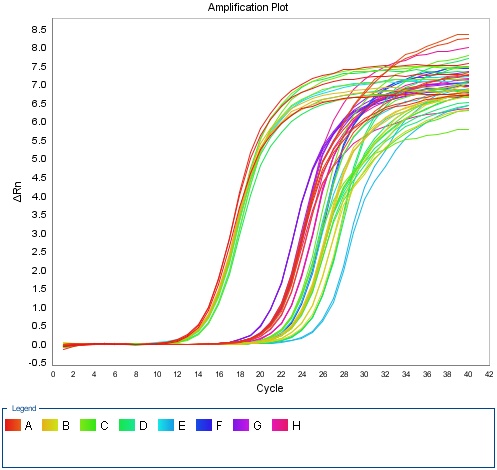


多样性扩增图

7.1溶解曲线


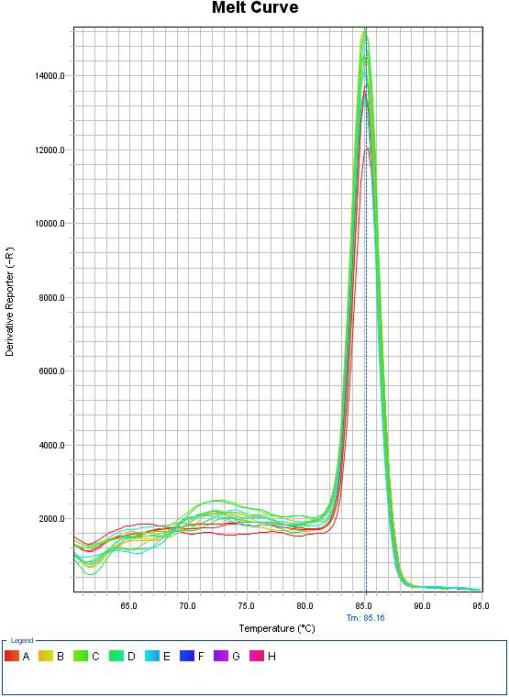

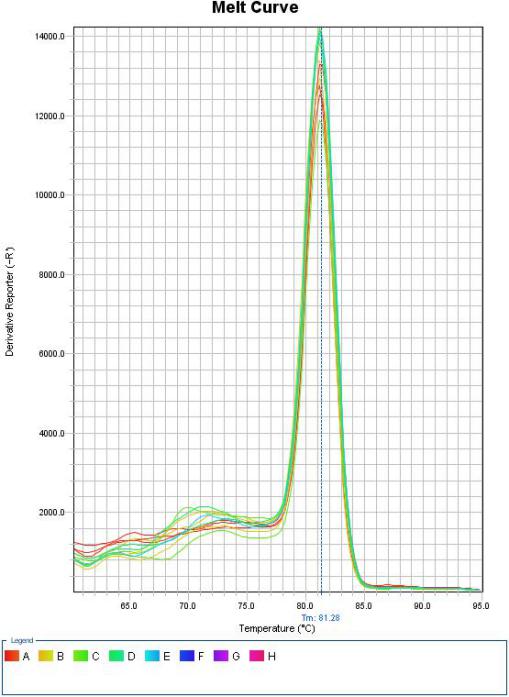


GAPDH MOF


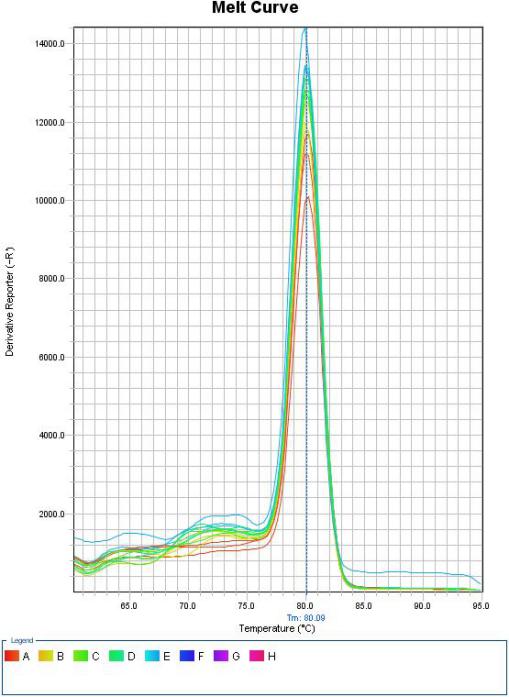

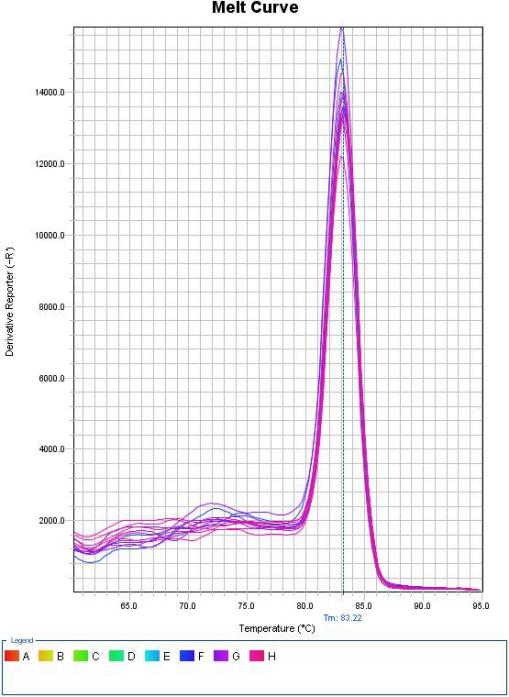


Sirt1 Beclin1

7.2 标准曲线


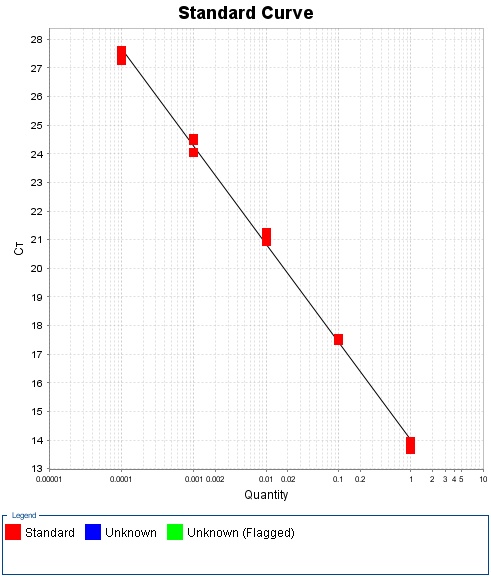


|  | std1 | std2 | std3 | std4 | std5 |
| --- | --- | --- | --- | --- | --- |
| Quantity | 1 | 0.1 | 0.01 | 0.001 | 1.00E-04 |
| Cт | 13.931483 | 17.522161 | 20.932955 | 24.029856 | 27.612341 |
|  | 13.798659 | 17.466747 | 21.245022 | 24.534435 | 27.538734 |
|  | 13.658651 | 17.547737 | 21.091108 | 24.452158 | 27.248682 |
| Cт Mean | 13.796265 | 17.512215 | 21.089695 | 24.338816 | 27.466586 |

| slope | -3.518 |
| --- | --- |
| Y-Inter | 17.131 |
| R2 | 0.998 |

7.3 数据统计结果

CT值表示每个反应管内的荧光信号到达设定的域值时所经历的循环数。

△Ct=目的基因Ct-内参基因Ct

实验组与正常组相对表达量=2^(-⊿⊿Ct)^

1. hMOF检测结果

| 实验分组 | A | B | C | D | E |
| --- | --- | --- | --- | --- | --- |
| GAPDH | 15.17676258 | 15.4835701 | 15.56959534 | 15.51417065 | 15.86914444 |
|  | 15.02522564 | 15.37739277 | 15.64961624 | 15.94691372 | 15.92791557 |
|  | 15.06045246 | 15.4029665 | 15.91215134 | 15.57910538 | 15.52011681 |
| 平均值 | 15.08748023 | 15.42130979 | 15.71045431 | 15.68006325 | 15.77239227 |
| hMOF | 25.29641724 | 22.75183678 | 24.15974579 | 24.34538345 | 23.81980858 |
|  | 25.3135128 | 22.6904068 | 23.86377579 | 24.28368835 | 23.60724487 |
|  | 25.19629326 | 23.14527321 | 23.68652229 | 24.19488449 | 23.68315773 |
| 平均值 | 25.2687411 | 22.86250559 | 23.90334796 | 24.2746521 | 23.70340373 |
| △CT | 10.18126087 | 7.441195806 | 8.192893651 | 8.594588852 | 7.931011454 |
| △△CT | 0 | -2.740065066 | -1.988367221 | -1.58667202 | -2.250249418 |
| 2^△△CT | 1 | 6.681004664 | 3.967876769 | 3.003556952 | 4.757650906 |

1. Sirt1检测结果

| 实验分组 | A | B | C | D | E |
| --- | --- | --- | --- | --- | --- |
| GAPDH | 15.17676258 | 15.4835701 | 15.56959534 | 15.51417065 | 15.86914444 |
|  | 15.02522564 | 15.37739277 | 15.64961624 | 15.94691372 | 15.92791557 |
|  | 15.06045246 | 15.4029665 | 15.91215134 | 15.57910538 | 15.52011681 |
| 平均值 | 15.08748023 | 15.42130979 | 15.71045431 | 15.68006325 | 15.77239227 |
| Sirt1 | 19.36750793 | 21.35570259 | 21.6005764 | 20.55184937 | 21.09487343 |
|  | 19.63938332 | 21.86233635 | 20.52991104 | 20.29973526 | 21.84516525 |
|  | 19.37361145 | 21.54227638 | 20.49734688 | 20.56834984 | 21.86201668 |
| 平均值 | 19.46016757 | 21.58677177 | 20.87594477 | 20.47331149 | 21.60068512 |
| △CT | 4.37268734 | 6.165461985 | 5.165490468 | 4.79324824 | 5.828292847 |
| △△CT | 0 | 1.792774645 | 0.792803129 | 0.4205609 | 1.455605507 |
| 2^△△CT | 1 | 0.288616434 | 0.577221472 | 0.747134092 | 0.364602029 |

1. Beclin1检测结果

| 实验分组 | A | B | C | D | E |
| --- | --- | --- | --- | --- | --- |
| GAPDH | 15.17676258 | 15.4835701 | 15.56959534 | 15.51417065 | 15.86914444 |
|  | 15.02522564 | 15.37739277 | 15.64961624 | 15.94691372 | 15.92791557 |
|  | 15.06045246 | 15.4029665 | 15.91215134 | 15.57910538 | 15.52011681 |
| 平均值 | 15.08748023 | 15.42130979 | 15.71045431 | 15.68006325 | 15.77239227 |
| Beclin1 | 24.14759827 | 23.47353745 | 23.16475716 | 22.26475716 | 23.62284546 |
|  | 24.24298363 | 23.63930168 | 22.28078651 | 22.28078651 | 23.43663216 |
|  | 24.22541046 | 23.78124695 | 22.3022213 | 22.3022213 | 23.26650505 |
| 平均值 | 24.20533079 | 23.63136202 | 22.58258832 | 22.28258832 | 23.44199422 |
| △CT | 9.117850558 | 8.210052236 | 6.872134018 | 6.602525075 | 7.669601949 |
| △△CT | 0 | -0.907798322 | -2.24571654 | -2.515325483 | -1.448248609 |
| 2^△△CT | 1 | 1.876180099 | 4.742726058 | 5.717266271 | 2.728765857 |

1. **ChIP检测H4K16ac在自噬靶基因Beclin1启动子区的结合**

**5实验结果**

**结果分析：**通过50*2^(Adjusted input - Ct (IP)计算得到的值比较。阴性对照（IgG）与目的基因组比较，目的基因组值显著高于阴性对照（IgG）值，说明抗体靶蛋白与目的基因有结合。

1. 正常对照组

|  | Target 1 | Target 2 | Target 3 |
| --- | --- | --- | --- |
| Input | 29.87848854 | 29.64103508 | 29.93549538 |
|  | 30.34075165 | 29.00311661 | 30.11222458 |
|  | 29.95612907 | 28.84175873 | 30.36875153 |
| 平均CT | 30.05845642 | 29.16197014 | 30.13882383 |
| Adjusted input | 24.414456421 | 23.517970139 | 24.494823827 |
| IGG | 36.62464905 | 34.94810486 | 35.55983162 |
|  | 36.608181 | 34.80747223 | 35.52003288 |
|  | 36.22066498 | 34.96902466 | 35.93954659 |
| 平均CT | 36.48449834 | 34.90820058 | 35.67313703 |
| 50*2^(Adjusted input - Ct (IGG) | 0.01 | 0.02 | 0.02 |
| IP-1（正常对照组） | 28.71972275 | 28.7133522 | 28.50532341 |
|  | 28.65252686 | 28.49147987 | 28.93323517 |
|  | 28.5254097 | 28.20375061 | 28.15113068 |
| 平均CT | 28.6325531 | 28.46952756 | 28.52989642 |
| 50*2^(Adjusted input - Ct (IP) | 2.69 | 1.62 | 3.05 |
|  |  |  |  |
|  | Target 1 | Target 2 | Target 3 |
| IP | 2.69 | 1.62 | 3.05 |
| IGG | 0.01 | 0.02 | 0.02 |

1. MCAO模型组

|  | Target 1 | Target 2 | Target 3 |
| --- | --- | --- | --- |
| Input | 28.95848656 | 28.44287872 | 29.2349453 |
|  | 29.62054062 | 29.14875412 | 29.50560188 |
|  | 28.40724564 | 28.85738754 | 30.05684471 |
| 平均CT | 28.99542427 | 28.81634013 | 29.59913063 |
| Adjusted input | 23.351424271 | 23.172340129 | 23.95513063 |
| IGG | 35.56567001 | 36.12789154 | 36.13332939 |
|  | 36.61661911 | 35.42409134 | 36.58718872 |
|  | 36.55315399 | 35.55209351 | 36.54169655 |
| 平均CT | 36.24514771 | 35.7013588 | 36.42073822 |
| 50*2^(Adjusted input - Ct (IGG) | 0.01 | 0.01 | 0.01 |
| IP-2（模型组） | 28.3789978 | 28.7133522 | 28.1216774 |
|  | 28.83239365 | 28.49147987 | 28.81134605 |
|  | 28.8650074 | 28.20375061 | 28.8038578 |
| 平均CT | 28.69213295 | 28.46952756 | 28.57896042 |
| 50*2^(Adjusted input - Ct (IP) | 1.23 | 1.27 | 2.03 |
|  |  |  |  |
|  | Target 1 | Target 2 | Target 3 |
| IP | 1.23 | 1.27 | 2.03 |
| IGG | 0.01 | 0.01 | 0.01 |

1. 模型+电针组

|  | Target 1 | Target 2 | Target 3 |
| --- | --- | --- | --- |
| Input | 27.99578094 | 27.25253677 | 28.50397301 |
|  | 28.74814415 | 27.45460701 | 28.36572266 |
|  | 27.08508492 | 27.1960144 | 28.37309647 |
| 平均CT | 27.94300334 | 27.30105273 | 28.41426404 |
| Adjusted input | 22.299003337 | 21.657052729 | 22.770264043 |
| IGG | 37.0183754 | 35.36834335 | 36.11170006 |
|  | 36.64121246 | 35.4626503 | 36.94902611 |
|  | 36.62347031 | 36.55257416 | 36.4667778 |
| 平均CT | 36.76101939 | 35.7945226 | 36.50916799 |
| 50*2^(Adjusted input - Ct (IGG) | 0.00 | 0.00 | 0.00 |
| IP-3（模型+电针组） | 28.49173927 | 28.73768425 | 28.55294991 |
|  | 28.19754028 | 28.64545822 | 28.50038147 |
|  | 28.80574989 | 28.96329498 | 28.9480896 |
| 平均CT | 28.49834315 | 28.78214582 | 28.66714032 |
| 50*2^(Adjusted input - Ct (IP) | 0.68 | 0.36 | 0.84 |
|  |  |  |  |
|  | Target 1 | Target 2 | Target 3 |
| IP | 0.68 | 0.36 | 0.84 |
| IGG | 0.00 | 0.00 | 0.00 |

（4）模型+电针+hMOF siRNA干预组

|  | Target 1 | Target 2 | Target 3 |
| --- | --- | --- | --- |
| Input | 27.3747921 | 27.51180267 | 27.9062748 |
|  | 27.94935989 | 27.85268211 | 27.83244133 |
|  | 28.18231964 | 27.89804077 | 28.50686836 |
| 平均CT | 27.83549054 | 27.75417519 | 28.0818615 |
| Adjusted input | 22.191490545 | 22.110175186 | 22.437861496 |
| IGG | 36.38101196 | 36.12081146 | 32.47367859 |
|  | 35.81535721 | 34.85561371 | 33.38418579 |
|  | 36.71992493 | 37.07054901 | 34.25456238 |
| 平均CT | 36.30543137 | 36.01565806 | 33.37080892 |
| 50*2^(Adjusted input - Ct (IGG) | 0.00 | 0.00 | 0.03 |
| IP-4（模型+电针+hMOF干预组） | 28.46502113 | 28.78886032 | 27.87703705 |
|  | 29.02816963 | 28.9828701 | 28.90427589 |
|  | 29.02401161 | 29.23933601 | 29.12059784 |
| 平均CT | 28.83906746 | 29.00368881 | 28.63397026 |
| 50*2^(Adjusted input - Ct (IP) | 0.50 | 0.42 | 0.68 |
|  |  |  |  |
|  | Target 1 | Target 2 | Target 3 |
| IP | 0.50 | 0.42 | 0.68 |
| IGG | 0.00 | 0.00 | 0.03 |

（5）模型+电针+Sirt1抑制剂组

|  | Target 1 | Target 2 | Target 3 |
| --- | --- | --- | --- |
| Input | 29.99523926 | 28.62857246 | 29.68465233 |
|  | 29.47725105 | 28.58568192 | 29.27009583 |
|  | 28.86466408 | 28.25535965 | 29.32857704 |
| 平均CT | 29.44571813 | 28.48987134 | 29.42777507 |
| Adjusted input | 23.801718129 | 22.845871343 | 23.783775065 |
| IGG | 33.77920151 | 32.38001633 | 33.9729805 |
|  | 33.09678268 | 32.65357208 | 33.93563461 |
|  | 33.14302063 | 32.41573715 | 33.84386826 |
| 平均CT | 33.33966827 | 32.48310852 | 33.91749446 |
| 50*2^(Adjusted input - Ct (IGG) | 0.07 | 0.06 | 0.04 |
| IP-5（模型+电针+Sirt1抑制剂组） | 28.45239067 | 28.72086143 | 28.87650108 |
|  | 28.86977768 | 28.41922569 | 28.96685028 |
|  | 29.22020912 | 29.24781036 | 29.05624771 |
| 平均CT | 28.84745916 | 28.79596583 | 28.96653303 |
| 50*2^(Adjusted input - Ct (IP) | 1.51 | 0.81 | 1.38 |
|  |  |  |  |
|  | Target 1 | Target 2 | Target 3 |
| IP | 1.51 | 0.81 | 1.38 |
| IGG | 0.07 | 0.06 | 0.04 |
